# hiPSC-Derived Neurons Provide a Robust and Physiologically Relevant *In Vitro* Platform to Test Botulinum Neurotoxins

**DOI:** 10.3389/fphar.2020.617867

**Published:** 2021-01-14

**Authors:** Juliette Duchesne De Lamotte, Sylvain Roqueviere, Hélène Gautier, Elsa Raban, Céline Bouré, Elena Fonfria, Johannes Krupp, Camille Nicoleau

**Affiliations:** ^1^ IPSEN Innovation, Les Ulis, France; ^2^ IPSEN Bioinnovation, Abingdon, United Kingdom

**Keywords:** botulinum neurotoxins, *in vitro* translational models, human induced pluripotent stem cells, motor neurons, neuromuscular junction

## Abstract

Botulinum neurotoxins (BoNTs) are zinc metalloproteases that block neurotransmitter release at the neuromuscular junction (NMJ). Their high affinity for motor neurons combined with a high potency have made them extremely effective drugs for the treatment of a variety of neurological diseases as well as for aesthetic applications. Current *in vitro* assays used for testing and developing BoNT therapeutics include primary rodent cells and immortalized cell lines. Both models have limitations concerning accuracy and physiological relevance. In order to improve the translational value of preclinical data there is a clear need to use more accurate models such as human induced Pluripotent Stem Cells (hiPSC)-derived neuronal models. In this study we have assessed the potential of four different human iPSC-derived neuronal models including Motor Neurons for BoNT testing. We have characterized these models in detail and found that all models express all proteins needed for BoNT intoxication and showed that all four hiPSC-derived neuronal models are sensitive to both serotype A and E BoNT with Motor Neurons being the most sensitive. We showed that hiPSC-derived Motor Neurons expressed authentic markers after only 7 days of culture, are functional and able to form active synapses. When cultivated with myotubes, we demonstrated that they can innervate myotubes and induce contraction, generating an *in vitro* model of NMJ showing dose-responsive sensitivity BoNT intoxication. Together, these data demonstrate the promise of hiPSC-derived neurons, especially Motor Neurons, for pharmaceutical BoNT testing and development.

## Introduction

Botulinum neurotoxins (BoNTs) are zinc metalloproteases produced by the Gram-positive anaerobic bacterium Clostridium botulinum and have a high potency for inhibition of neurotransmitter release from peripheral neurons, in particular Acetylcholine release at the Neuro Muscular Junction thereby inducing muscle weakness (Simpson, 1980; Foster, 2014a; Foster, 2014b; Rossetto et al., 2014; Rummel, 2015). While they are the causative agent of botulism, a disease characterized by a progressive descending flaccid paralysis, their high affinity for motor neurons combined with a high potency have made them extremely valuable drugs for treatment of a variety of neurological diseases as well as for aesthetic applications (Foster, 2014a; Foster, 2014b).

BoNT is synthesized as a 150 kDa single chain and then cleaved into a 100 kDa heavy chain linked by a disulfide bridge to a 50 kDa light chain (Rossetto et al., 2014). The heavy chain is responsible for binding the toxin to presynaptic surface receptors of cholinergic nerve terminals and promotes light-chain translocation across the endosomal membrane after endocytosis (Beard, 2014). The light chain acts as a zinc endopeptidase with proteolytic activity located at the N-terminal end (Montal, 2010; Rossetto et al., 2014; Fonfria, 2018).

Seven antigenically distinct but structurally similar serotypes of botulinum neurotoxin (BoNT) have been reported so far, from BoNT/A to G (Foster, 2014a; Rossetto et al., 2014; Peck et al., 2017). More recently, an eighth serotype has been identified at the protein sequence level, BoNT/X (Zhang et al., 2017).

BoNTs exhibit neuronal intoxication through a multistep process: 1) binding, 2) internalization, 3) translocation, 4) cleavage. Although all botulinum toxin serotypes block the release of acetylcholine from nerve endings by affecting the soluble N-ethylmaleimide-sensitive fusion protein attachment protein receptor (SNARE), complex essential for acetylcholine release, they differ in their specificity for surface receptors as well as their specificity for individual SNARE proteins.

The first step in the BoNT intoxication process involves binding to a ganglioside receptor present on the presynaptic membrane, followed by binding to a protein receptor of the lumen of synaptic vesicles (Montecucco, 1986; Rummel, 2016). Gangliosides are a group of glycosphingolipid molecules with sialic acid moieties linked to oligosaccharides, particularly abundant in neuronal membranes (Salinas et al., 2010). Their biosynthesis involves sequential activities of distinct membrane-spanning sialyltransferases and glycosyltransferases that regulate the number and position of their sialic acids. Amongst the large family of gangliosides, GM2, GD1a, GD1b and GT1b are the most abundant in the nervous system, with GT1b and GD1 being the preferred forms recognized by BoNTs (Rummel, 2016; Hamark et al., 2017). Ganglioside binding accumulates the toxin on the nerve terminal surface and thus facilitates its interaction with a protein receptor (Rummel, 2016; Hamark et al., 2017).

BoNT/A,/E and/F bind to synaptic vesicle protein SV2 (Dong, 2006; Dong et al., 2008) ; BoNT/B and/G bind to synaptotagmin 1 and 2 (Syt 1, 2) (Rummel et al., 2007; Stenmark et al., 2010; Willjes et al., 2013); BoNT/C binds to two gangliosides to gain cellular entry (Karalewitz et al., 2012) ; BoNT/D may use SV2 as a receptor (Peng et al., 2011; Peng et al., 2012) and cell binding modalities of BoNT/X have yet to be explored (Zhang et al., 2017). Furthermore, while BoNT/A can bind to all three isoforms (SV2A, SV2B, and SV2C), BoNT/E binds only to the glycosylated forms of SV2A and SV2B (Dong, 2006; Dong et al., 2008; Benoit et al., 2014).

After binding, BoNT is internalized inside synaptic vesicles. The acidification of these vesicular lumens induces the protonation and not-well understood conformational changes of BoNT that lead to membrane translocation. The Light Chain is then released into the cytosol by the action of the thioredoxin reductase – thioredoxin system (TRXR-TRX) which reduces the interchain disulphide bond.

Once in the cytosol, the Light Chain cleaves specific components of the SNARE complex: BoNT/A and BoNT/E cleave synaptosomal-associated protein 25 (SNAP25); BoNT/B, BoNT/D, BoNT/F, BoNT/G and BoNT/X cleave synaptobrevin (VAMP); and BoNT/C cleaves both SNAP25 and syntaxin (Blasi et al., 1993; Schiavo et al., 1995; Foran et al., 1996).

The number of indications treated with BoNTs has been constantly increasing since their first clinical use in 1980 (Fonfria, 2018). Because of their fascinating modular molecular architecture, their high potency and the emergence of recombinant production methods offering the possibility to modify and engineer them as required, the therapeutic potential of these molecules for a plethora of indications is large (Chaddock, 2012; Fonfria et al., 2018b; Pons et al., 2019). Current *in vitro* models used for the development of BoNTs include rodent primary cultures (Keller et al., 2004; Restani et al., 2012) and immortalized cell lines (Purkiss et al., 2001; Tegenge et al., 2012). Rodent primary cultures have several limitations such as a poor reproducibility and stability over time; they also raise important ethical considerations. In addition, cross species differences have been reported in some BoNT receptors or SNARE BoNT substrates (Peng et al., 2012; Strotmeier et al., 2012; Elliott et al., 2019), limiting the translatability of data from rodent primary cells as well as immortalized rodent cell lines. Human neuronal immortalized cell lines partially overcome this hurdle, but still have limitations concerning their physiological relevance (Gordon et al., 2013).

Since their first generation in 2008 (Takahashi et al., 2007; Yu et al., 2007), human induced pluripotent stem cells (hiPSCs) have been widely used in disease modeling, drug discovery and cell therapy development for a broad spectrum of human diseases including neurological disorders (Shi et al., 2017; Little et al., 2019; Rowe and Daley, 2019) and have also shown great potential for predictive toxicology (Liu et al., 2017). The establishment of robust methods for differentiating hiPSCs into various neuronal subtypes (Kriks et al., 2011; Marteyn et al., 2011; Kirkeby et al., 2012; Nicoleau et al., 2013; Gribaudo et al., 2019) has revolutionized their usage in drug discovery in neurology (Marteyn et al., 2011; Egawa et al., 2012; Charbord et al., 2013).

With regard to BoNT research and drug discovery, the most commonly used hiPSC-derived neuronal model is a GABAergic neuronal model (Whitemarsh et al., 2012; Pellett et al., 2015a; Pellett et al., 2015b; Elliott et al., 2019). However, as GABA is an inhibitory neurotransmitter, the usage of GABAergic neurons for functional assays remains challenging and might demand, for example, the usage of radioactive elements (Elliott et al., 2019) which requires trained staff and special authorization. Furthermore, even though all the proteins necessary for the toxin to be active are expressed by GABAergic neurons, this model does not represent the most physiologically relevant cell type for BoNT activity, i.e. cholinergic motor neurons.

We have recently highlighted the potential of three additional hiPSC-derived neuronal models, including a Motor Neuron model, to provide a sensitive platform for studying BoNT intoxication mechanisms and for BoNT potency determination (Nicoleau et al., 2018a; Nicoleau et al., 2018b). The potential of hiPSC-derived Motor Neurons for BoNT testing has been confirmed more recently by two studies (Pellett et al., 2019; Schenke et al., 2020). However, most assays using hiPSC-derived neurons for BoNT study and testing used so far have been biochemical assays focusing on the quantification of one specific BoNT target which does not allow the comparison of BoNTs cleaving different SNARE. The development of functional assays incorporating hiPSC-Motor Neurons would overcome this limitation and offer the possibility to study, test and compare different BoNT serotypes in physio-relevant systems. Several protocols for differentiating hiPSCs into Motor neurons are now available for the generation of functional Motor Neurons that can be incorporated in physiological systems such as the Neuro Muscular Junction (Maury et al., 2015; Puttonen et al., 2015; Steinbeck et al., 2016; Bianchi et al., 2018; Santhanam et al., 2018).

In this study, we have thoroughly characterized and compared four different neuronal models derived from hiPSCs by analyzing their gene and protein expression profiles and by assessing their sensitivity to two different BoNT subtypes, BoNT/A and BoNT/E. The most clinically relevant of these models, hiPSC derived Motor Neurons, expressed markers of authentic Motor Neurons and were highly sensitive to intoxication by both serotype A and E BoNTs. We assessed the physiological and functional features of hiPSC-derived Motor neurons cultures by performing RNA sequencing and conducting electrophysiological studies and calcium mobilization analyses. And finally, we have exploited functional characteristics and innervation potential of these hiPSC-derived Motor neurons in presence of human myotubes. The coculture system led to the establishment of a model of NMJ sensitive to BoNT highlighting the high promise and potential of hiPSC-derived Motor Neurons to enhance the physiological relevance of preclinical data on BoNTs.

## Material and Methods

### Culture of hiPSC-Derived Neuronal Models

Frozen hiPSC-derived GABAergic Neurons, Glutamatergic Neurons and Motor Neurons (iCell GABA N, iCell GLUTA N and iCell Motor Neurons respectively) were provided by FujiFilm Cellular Dynamics International (FCDI, Madison, WI) and Peripheral neurons (Peri.4U) were provided by Ncardia (Gosselies, Belgium). Frozen cells were thawed and plated according to the provider instructions and at the recommended density (125 × 10^3^ cells/cm^2^ for iCell GABA N, 100 × 10^3^ cells/cm^2^ for Motor Neurons, 150 × 10^3^ cells/cm^2^ for Peri.4U and 200 × 10^3^ cells/cm^2^ for iCell GLUTA N) into 24-well plates (TPP, Trasadingen, Switzerland) in a volume of 500 µL/well or into 96-well plates (TPP or Greiner Bio-One International GmbH, Kremsmünster, Austria) in a volume of 100 µl/well depending on the experiment. Each culture type was maintained in the medium recommended at 37°C, 5% CO_2_ for the indicated time. A 50%–75% medium change was realized every 2–3 days as recommended by suppliers. Cultures were observed regularly using an Olympus microscope (CKX53, Olympus Life Science Solutions, Waltham, MA).

### RNA Extraction and Quantitative Reverse Transcriptase Chain Reaction (qRT-PCR)

hiPSC-derived neurons were plated onto 24-well plates at the recommended density. Total RNA from two wells was isolated with the PicoPure RNA Isolation Kit (Applied Biosystems, Foster City, CA) according to the manufacturer’s instructions. Human adult total brain or human total Spinal Cord RNA (BioChain Institute Inc., Newark, CA) was used as positive control. cDNA was generated from 0.5 µg of RNA with High Capacity cDNA Reverse Transcription kit (Applied Biosystems). Quantitative real time-polymerase chain reactions (QRT-PCRs) were performed with the TaqMan™ Universal PCR Master Mix (Applied Biosystems) and the following TaqMan™ Human Gene Expression Assays: SNAP25 (Hs00938962_m1); SNAP23 (Hs00187075_m1); STX1A (Hs00270282_m1); STX1B (Hs01041315_m1); STX2 (Hs00984922_m1); VAMP1 (Hs01042310_m1); VAMP2 (Hs00360269_m1); VAMP3 (Hs00922166_m1); VAMP4 (Hs01002031_m1); VAMP5 (Hs01105383_g1); YKT6 (Hs00559911_g1); VAMP7 (Hs00194568_m1); VAMP8 (Hs00186809_m1); SYT1 (Hs00194572_m1); SYT2 (Hs00980604_m1); SV2A (Hs01059458_m1); SV2B (Hs00208178_m1); SV2C (Hs00392676_m1); ST3GAL2 (Hs00199480_m1); ST3GAL3 (Hs00544033_m1); B4GALT6 (Hs00191135_m1); UGCG (Hs00916612_m1) ; ST3GAL5 (Hs01105377_m1); ST8SIA1 (Hs01124289_m1); B4GALNT1 (Hs01110791_g1); B3GALT4 (Hs00534104_s1); ST8SIA5 (Hs00203298_m1); ST6GALNAC5 (Hs00229612_m1); TXN (Hs01555214_g1); TXNR (Hs00917067_m1) and GAPDH (Hs03929097_g1). Quantification was performed at a threshold detection line (Ct value). The Ct of each target gene was normalized to GAPDH housekeeping gene.

### Immunostaining

For immunostaining, hiPSC-derived neurons were plated into 96-well plates. 15 days after plating, neurons were fixed with 4% paraformaldehyde for 30 min at 4°C. Cultures were washed twice with PBS (Thermo Fisher Scientific, Waltham, MA) and then permeabilized and blocked with PBS containing 2% BSA (A9647, Sigma-Aldrich, Saint-Louis, MO) and 0.1% Triton (X100, Sigma-Aldrich) for 30 min at RT. The following primary antibodies were incubated overnight at 4°C in PBS 2% BSA, 0.1% Triton: MAP2 (M1406, Sigma-Aldrich), TUJ1 (Ab107216, Abcam, Cambridge, United Kingdom ; Ab41489, Abcam ; BLE801202, BioLegend, San Diego, CA, USA), SMI-32 (ab8135, Abcam), SYN (NB300-104, Novus Biologicals, Centennial, CO, USA), SNAP25 (S9684, Sigma-Aldrich), GABA (A0310, Sigma-Aldrich), PRPH (sc-377093, Santa Cruz Biotechnology Inc., Dallas, TX), vGlut2 (135403, Synaptic Systems GmbH, Göttingen, Germany), Isl1 (GT15051, Neuromics Inc., Edina, MN, USA), SV2A (119002, Synaptic Systems), VAMP2 (ab181869, Abcam), SAA (A7811, Sigma-Aldrich), Desmin (ab80503, Abcam), GT1b (MAB5608, Merck Millipore, Burlington, MA), GD2 (Ab68456, Abcam).

Cultures were then washed with PBS and stained with the corresponding secondary antibodies anti-rabbit, anti-mouse, anti-goat, anti-chicken, as well as DAPI, MF20-AF488 (53-6503-82, Thermo Fisher Scientific) and αBTX (Alexa Fluor 647, B35450, Thermo Fisher Scientific ; Rhodamine, T0195, Sigma-Aldrich) for 1 h 30 at RT.

Cultures were washed again with PBS and then visualized using either the ImageXpress Micro Confocal High-Content Imaging System (Molecular Devices, San José, CA, USA) at 20X magnification or the Eclipse Ti-E inverted fluorescence microscope (Nikon Instruments Inc., Tokyo, Japan) coupled to an ORCA Flash 4 LT camera (Hamamatsu Photonics, Hamamatsu City, Japan) at 20X and 40X magnifications.

### Botulinum Neurotoxins Treatment

BoNT serotype A and E were manufactured by Ipsen as previously described (Hooker et al., 2016; Fonfria et al., 2018a; Pons et al., 2019). Inactive BoNT/A (BoNT/A (0)) was produced by point mutations E224Q, H227Y in BoNT/A (Fonfria et al., 2018a). All BoNTs were purified and activated to more than 91%. For assessing the effect of Botulinum Neurotoxin A and E, hiPSC-derived neurons were plated in 96-well plate and exposed to the indicated doses of Botulinum toxin A or E in 100 µl of culture medium. For each experiment, each dose of BoNT was tested in triplicate and a negative control (medium without toxin) was always included. After 24 h the medium containing the toxin was removed, cells were washed with 200 µl PBS and lysed in 100 µl MPER. For each BoNT, each experiment was replicate in triplicate.

### SNAP25 Cleavage Assay by Western Blot

All samples were resolved by SDS-PAGE (Invitrogen, Carlsbad, CA) and transferred onto nitrocellulose membranes. Membranes were blocked with 5% nonfat milk in 0.1% PBS-T (phosphate buffered saline with Tween-20) and probed with an anti-SNAP25 antibody (1/2000; S9684, Sigma-Aldrich). Immunoreactive bands were detected using horseradish peroxidase–conjugated secondary anti-rabbit (1/2000; A6154, Sigma-Aldrich) antibodies and SuperSignal West Dura ECL substrate (Thermo Fisher Scientific). Bands were visualized on a Pxi4 imaging system using GeneSys image acquisition software (Syngene, Bangalore, Karnataka, India), and intensities of the total form and cleaved form of SNAP25 were measured with GeneTools (Philomath, OR, USA).

### RNASeq Analysis of hiPSC-Derived Motor Neurons and Human Spinal Cord

For the quantification of gene expression, libraries were prepared with TruSeq Stranded mRNA kit protocol according to supplier recommendations. After purification of PolyA containing mRNA molecules using poly-T oligo attached magnetic beads from 1 µg total RNA, a fragmentation using divalent cations under elevated temperature to obtain approximately 300 bp pieces was performed, followed by double strand cDNA synthesis and finally Illumina adapters ligation and cDNA library amplification by PCR for sequencing. Sequencing was then carried out on paired-end 75 b of Illumina NovaSeq. For the unsupervised analysis, we used the Bioconductor edgeR package to import raw counts into R statistical software and compute normalized log2 CPM (counts per millions of mapped reads) using the TMM, weighted trimmed mean of M-values, as normalization procedure. The normalized expression matrix from the 1,000 most variant genes (based on standard deviation) and from a custom list of genes were used to classify the samples according to their gene expression patterns using principal component analysis (PCA), hierarchical clustering and consensus clustering. We used FactoMineR:: PCA function to perform the PCA with “ncp = 10,scale.unit = FALSE” parameters. Hierarchical clustering was performed with stats:hclust function (with euclidean distance and ward.D method). For the differential expression analysis, we used the Bioconductor edgeR package to import raw counts into R statistical software. We then used the Bioconductor limma package to test for differential expression using the voom transformation. We only tested genes expressed in at least one sample (FPKM >= 0.1) to improve the statistical power of the analysis. We applied a *q*-value threshold of <= 0.05 and a minimum fold change of 1.2 to define differentially expressed genes. For the pathway enrichment analysis, we used hypergeometric tests to identify gene sets of the Gene Ontology collection from MSigDB v7.0 database overrepresented among the lists of up- or down-regulated genes, correcting for multiple testing with the Benjamini-Hochberg procedure.

### Measurements of Ca^2+^ Transient

iCell Motor Neurons (FCDI) were cultured in 96-well plates at a density of 90 × 10^3^ cells/cm^2^ for 28 days. For Ca^2+^ dynamics experiments, 28 days old-cells were loaded for 15 min with the calcium indicator Cal520-AM (2 µM, Abcam); previously reconstituted in dimethylsulfoxide (DMSO, Sigma-Aldrich) in loading buffer composed of HBSS Ca^2+^/Mg^2+^ (Thermo Fisher Scientific), 20 mM Hepes (Thermo Fisher Scientific), 2 mM NaOH (Sigma-Aldrich). Then cells are washed three times with recording buffer composed of HBSS without Ca^2+^/Mg^2+^ (Thermo Fisher Scientific), 20 mM Hepes (Thermo Fisher Scientific), 2 mM NaOH (Sigma-Aldrich), 2 mM CaCl_2_ (Sigma-Aldrich). Cells were then placed in recording buffer and challenged with products by the addition of 0.1% DMSO considered as vehicle, or 1 µM Tetrodotoxin (TTX, Tocris Bioscience, Bristol, United Kingdom), previously reconstituted in DMSO. To visualize Ca^2+^ transient, a live imaging system with an Evolve EMCCD camera (Zeiss, Oberkochen, Germany) coupled to a Spinning Disk system (Nipkow, CSU-X1M 5000, Zeiss) was used, and timelapse imaging (3 min film with time interval of 500 ms, 20X magnifications) was performed under physiological conditions (37°C and 5% CO2). A recording of Ca^2+^ oscillations with FDSS 6000 (Hamamatsu) imaging-based plate reader (10 min with time interval of 700 milliseconds) was also performed. Analyses of the Ca^2+^ oscillations frequency for each well were performed using the FDSS Waveform analysis software (Hamamatsu).

### Patch Clamp Analysis of Human hiPSC-Derived Motor Neurons in Monoculture and Coculture With Myotubes

**FIGURE 1:**
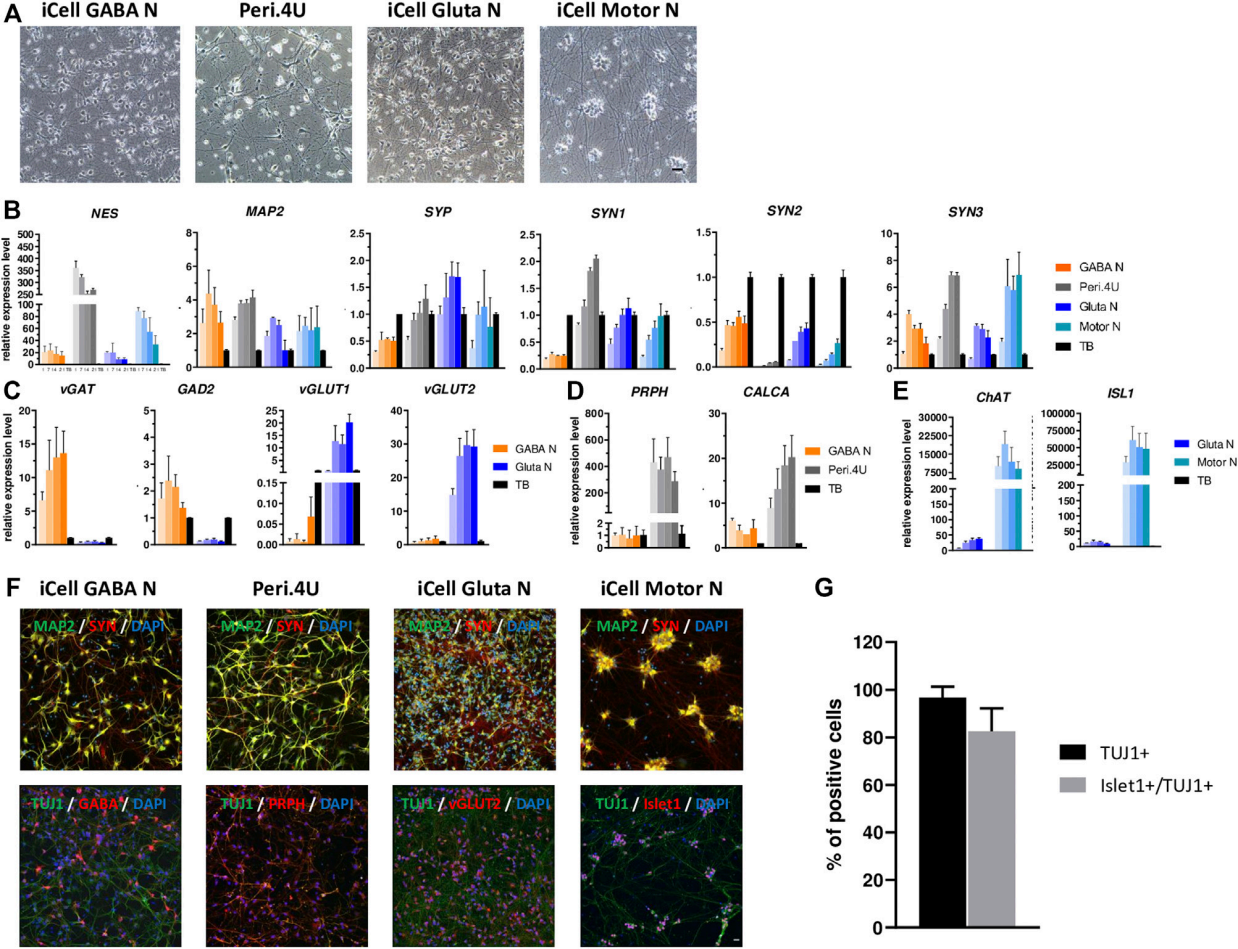
Characterization of multiple neuronal models derived from hiPSC. **(A)** Phase contrast images taken with a 20X objective at day 15 after thawing. Scale bar: 25 μm. **(B–E)** Time-course gene expression analysis of neuronal markers (B) and phenotypic markers **(C,E)** for the four models. Expression is normalized to GAPDH and to a control cDNA (human total brain: TB). For every model, each shade bar represents a different time point: 1 day, 7 days, 14 days and 28 days after thawing; 1 day being the lighter shade. **(F)** Immunolabelings at day 15 after thawing of markers for neurons (TUJ1 or MAP2), synapses (SYN), GABAergic neurons (GABA), peripheric neurons (PRPH), glutamatergic neurons (vGlut2) and motor neurons (Islet1). Nuclei are stained in blue (DAPI). Scale bar: 25 μm. **(G)** % of islet1+ cells amongst total cells and amongst neurons (TUJ1+).

Whole-cell patch clamp recordings were conducted at room temperature using an Axopatch 200B (Molecular Devices) connected to a 16-bit analogue-to-digital converter (Digidata 1,440, Molecular Devices). The pClamp 10.2 software package was used for data acquisition (sampling rate 20 kHz, low-pass eight-pole bessel filter 5 kHz) and analysis were done in Clampfit (Molecular Devices) or automated in Matlab (MathWorks, Natick, MA, USA) with custom-made scripts. Graphs were made in Graphpad Prism (GraphPad Software Inc., La Jolla, CA). All chemicals were purchased from Sigma-Aldrich^®^. Cells were perfused with bicarbonate-buffered solution containing (mM): 126 NaCl, 3.6 KCl, 1.5 MgSO_4_, 1.2 NaH_2_PO_4_, 26 NaHCO_3_, 0.5 CaCl_2_, 10 glucose, 305 mOsm, bubbled with 95%O_2_/5%CO_2_. Electrodes contained a solution comprising (mM): 135 Kgluconate, 5 NaCl, 1 CaCl_2_, 10 HEPES, 0.5 EGTA, 3 MgATP, 0.2 Na_2_GTP, 10 phosphocreatine-Mg, 2 KLucifer yellow, pH set to 7.3 with KOH, 299 mOsm. Series resistance was 5–25 MΩ (if higher, cells were excluded from the analysis) and electrode junction potentials (−15 mV) were corrected for offline. Cells were identified (Figure 1C) by their post-recording dye-fill morphology and confirmed by antibody labeling (Gautier et al., 2015) against Islet1 (5/10) and TUJ1 (10/10, monoculture). Only double positive cells were included in the analysis.

### Cocultures of Human Myotubes and Motor Neurons

Human immortalized myoblasts (AB1167c4, Institute of Myology, Paris, France) were seeded in Myoblast seeding medium on 96-well plates coated with Collagen I (Thermo Fisher Scientific) at a concentration of 70 × 10^3^ cells/cm^2^ and incubated at 37°C within a 5% CO_2_ environment. The myoblasts seeding medium was composed of 4 volume of DMEM high glucose GlutaMAX (Thermo Fisher Scientific), 1 volume of Medium 199 (Thermo Fisher Scientific), 20% FBS (Sigma-Aldrich) and 50 µg/ml gentamicin (Thermo Fisher Scientific) supplemented with 25 μg/ml fetuin (Sigma-Aldrich), 5 μg/ml recombinant human insulin (Thermo Fisher Scientific), 0.2 μg/ml dexamethasone (Sigma-Aldrich), 0.5 ng/ml recombinant human basic FGF (Thermo Fisher Scientific) and 5 ng/ml recombinant human EGF (Thermo Fisher Scientific). After 24 h, the myoblasts seeding medium was replaced with coculture medium composed of a mix between myoblasts differentiation medium and motor neurons growth medium (ratio respectively 1/3 and 2/3). The myoblasts differentiation medium is composed of DMEM high glucose GlutaMAX (Thermo Fisher Scientific), 50 µg/ml gentamicin (Thermo Fisher Scientific) and 10 µg/ml Insulin (Thermo Fisher Scientific). The motor neurons growth medium composition is based on the protocol developed by Martinat’s lab (Maury et al., 2015). iCell Motor Neurons (FCDI) were seeded at the density of 90 × 10^3^ cells/cm^2^, plated directly over the human myoblasts and incubated at 37°C with 5% CO_2_ for up to 15 days. A 50% medium change was realized once a week.

### Analysis of Myotube Contractions and Effect of Treatment

Human myotubes and Motor Neurons were cultured in 96-well plates for 15 days. For recording of contractions, cells were placed in a Spinning Disk microscope system (Zeiss) under physiological conditions (37°C and 5% CO_2_). Recording of contractions was performed in basal condition (before drugs addition) during 1min30 in phase contrast timelapse imaging (10X magnification) with time interval of 600ms, then after addition of 150 µM Tubocurarine (Sigma-Aldrich), 2 µM Tetrodotoxin (TTX, Tocris), BoNT/A and BoNT/A (0) during 1 min 30 in phase contrast timelapse imaging (10X magnification) with time interval of 600 ms at different timepoints. Analysis was performed with the open-source software tool MUSCLEMOTION (Sala et al., 2018) on ImageJ software, to quantify contractions according to instructions.

### Data Analysis

All data are expressed as individual data or as means ± SEM of *n* independent experiments. Dose-response curves were fitted by a four-parameter logistic equation, and the pEC_50_ was calculated. All data processing and statistical tests were done using GraphPad Prism version 7.04 (GraphPad Software Inc., La Jolla, CA).

Ordinary One Way of variance and Dunnett’s multiple comparisons: *p value<0.05 ; **p value <0.01; ***p value <0.001 and ****p < 0,0001.

## Results

### Morphologic and Phenotypic Characterization of Different hiPSC-Derived Neuronal Models

In order to assess and compare the potential of hiPSC-derived GABA-, Glutaminergic-, Peripheral - and Motor- Neurons for BoNT testing, we first performed a full time-course characterization of these models when maintained in culture for four weeks, based on morphological observation, gene expression analysis and immunostainings.

After 14 days in culture the four cryopreserved hiPSC-derived neuronal models each formed a dense network of neurites and axons (Figure 1A). While iCell GABA Neurons, Glutamatergic Neurons and Peri.4U had well-defined cellular bodies, iCell Motor Neurons tended to form cell body clusters (Figure 1A), as already reported by other studies on hiPSC-derived Motor Neurons (Maury et al., 2015; Steinbeck et al., 2016).

In order to characterize the neuronal maturity of the different neuronal cultures generated, we conducted a time-course gene expression analysis for different markers of neuronal progenitors, mature neurons and synaptic connections. Whatever the time in culture, Peri.4U neurons expressed a higher level for Nestin (*NES*), a marker for non-mature neurons, than the other three models and compared to human total brain, which served as a control (Figure 1B). The expression of *MAP2*, a marker for more mature neurons, was quite high compare to control, stable over time and comparable in the four models suggesting the four models acquired a neuronal maturity after 2 weeks in culture (Figure 1B). The expression of synaptic markers such as Synaptophysin (*SYP*) and Synapsin (*SYN1, SYN2* and *SYN3*) suggested the presence of synaptic connections in the different cultures of hiPSC-derived neurons respectively as soon as day 7 (Figure 1B).

To further determine the phenotypic identity of neurons present within the different cultures generated, we then performed a time-course gene expression analysis of markers specific for GABAergic, Glutamatergic, Peripheral and Motor neurons. As expected, when comparing expression level for iCell GABA Neurons and iCell Gluta Neurons, we showed that iCell GABAN expressed higher level of vesicular Gaba transporter (*vGAT*) and Glutamate decarboxylase 2 (*GAD2*) than iCell GlutaN, whereas iCell Gluta Neurons expressed higher level of Vesicular glutamate transporter 1 and 2 (*vGLUT1* and *vGLUT2*) than iCell GABA Neurons (Figure 1C) confirming the respective identity of iCell GABAergic- and iCell Glutamatergic- neurons. When comparing iCell GABA Neurons and Peri.4U, data showed that Peri.4U expressed higher level of expression for Peripherin (*PRPH*) and calcitonin related polypeptide alpha (*CALCA*) (Figure 1D) confirming the peripheral identity of Peri.4U. Finally, QrtPCR analysis demonstrated that iCell Motor Neurons expressed a high level of Choline Acetyl Transferase (*ChAT*) and islet1 (*ISL1*) compared to Gluta Neurons and TB confirming the identity of this model (Figure 1E).

To confirm the expression of some of key genes, immunostainings were conducted in all models. The expression for neuronal markers MAP2 and Neuron-specific class III beta-tubulin (TUJ1) as well as Synapsin, a marker for synapses, was confirmed in all the models at day 14 (Figure 1F). Furthermore, the expression of markers specific to each neuronal type, ie. GABA for iCell GABA Neurons, vGLUT2 for iCell Gluta N, Peripherin for Peri.4U and Islet1 for Motor Neurons was confirmed. In addition, the percentage of TUJ1-positive cells and Islet1 positive cells amongst TUJ1 expressing cells in iCell Motor Neurons cultures was estimated to 96.7 and 82.5% respectively confirming the high purity of this culture (Figure 1G).

Altogether our results show that the four neuronal models derived from hiPSC express high level of neuronal and synaptic markers as well as markers specific of their respective neuronal identity 1 week after thawing and that these models can be maintained in culture up to 4 weeks without loss of phenotype demonstrating the value of hiPSC-derived neuronal models compared to primary cells (Nicoleau et al., 2018b) or neuronal immortalized cell lines.

In order to determine whether hiPSC-derived neuronal models could be used for measuring BoNT activity, the expression of BoNT protein receptors, SNARE substrates, and proteins needed for the light chain translocation were measured by QrtPCR over a period of 4 weeks in all four hiPSC-derived neuronal cultures. In order to make the analysis as exhaustive as possible, genes encoding SNAREs cleaved by the newly discovered BoNT/X as well as *VAMP7* and *VAMP8* were included in the analysis in addition to the genes conventionally studied in the field of BoNTs.

**FIGURE 2:**
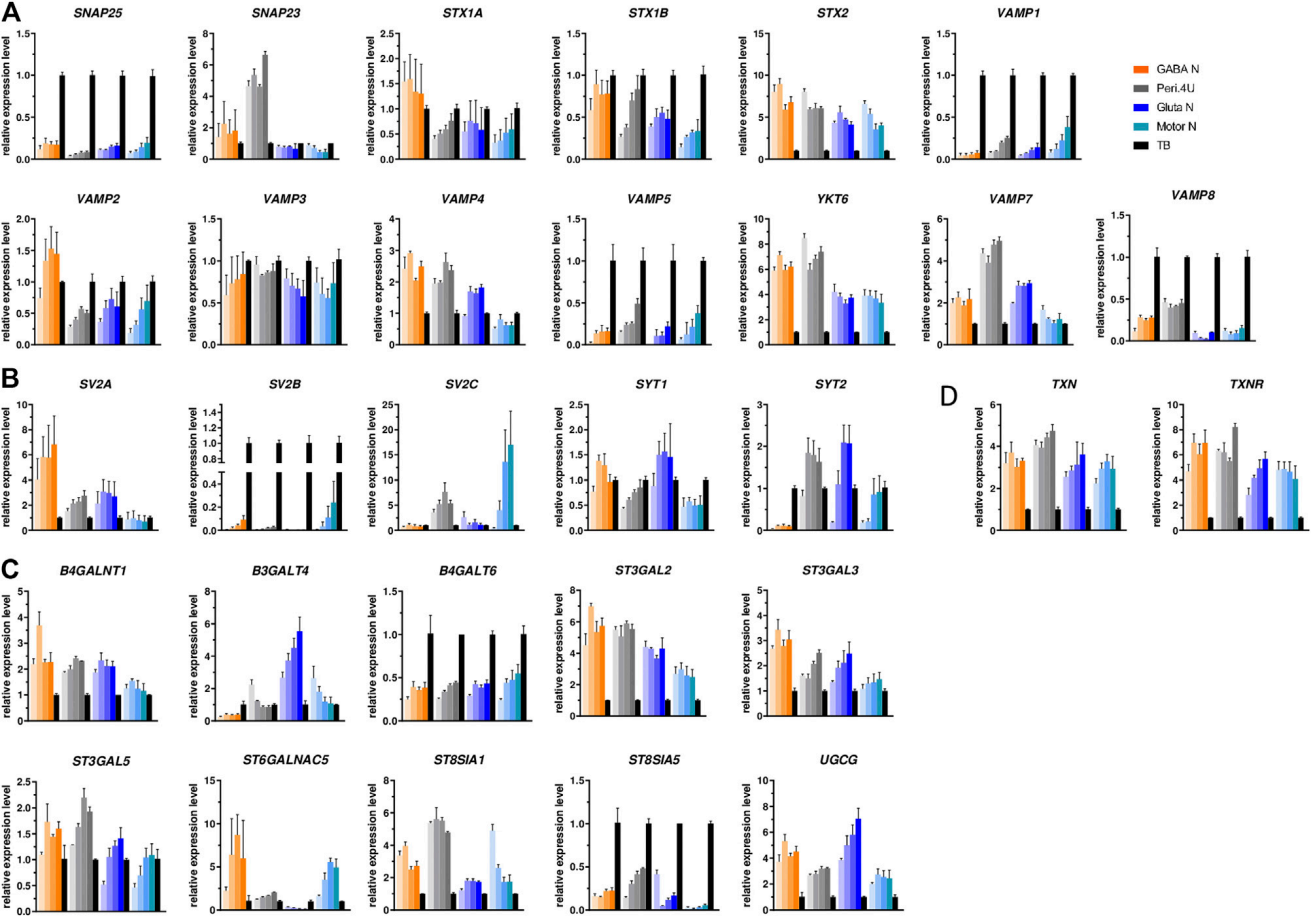
Expression of the different BoNT SNARE substrates, BoNT receptors and gangliosides. **(A–C)** Time-course gene expression analysis of various SNARE proteins **(A)**, BoNT proteic receptors **(B)** and enzymes involved in the biosynthesis of gangliosides **(C)** for the 4 models. For every model, each shade bar represents a different time point: 1 day, 7 days, 14 days and 28 days after thawing; 1 day being the lighter shade. Expression is normalized to GAPDH and to a control cDNA (human total brain: TB).

All SNARE, BoNT protein receptors, Thioredoxin and thioredoxin-reductase were expressed by the four hiPSC-derived models whatever the time in culture and up to 4 weeks. For the genes *SNAP25*, *STX1A*, *STX1B*, *VAMP3*, *VAMP5*, *SYT1*, *TNX* and *TNXR* the expression level was quite comparable when comparing the four models (Figure 2). For some other analyzed genes, differences in the level of expression among models were detected. GABA neurons express higher expression of *VAMP2* and *SV2A*, Peripheral neurons expressed higher level of *SNAP23* and *VAMP7* compared to other models. Also, these two models expressed higher level of *STX2*, *YKT6* and *VAMP8* compared to Glutamatergic neurons and Motor Neurons while *SYT2* was more highly expressed by Peripheral and glutamatergic neurons (Figures 2A,B). Motor Neurons express higher level of *VAMP1*, *SV2B* and *SV2C* and lower expression of *VAMP4* compared to other models (Figures 2A,B). As BoNT binds also to gangliosides, we assessed the expression of the 10 enzymes known to be involved in ganglioside biosynthesis. The expression of *ST3GAL5*, *B4GALT6* and *ST6GALNAC5* was comparable in all four models (Figure 2C). *ST3GAL2*, *ST3GAL3* were more highly expressed by Motor Neurons and on the contrary, B4GALNT1 and ST8SIA5 were less expressed by Motor Neurons (Figure 2C). The expression of *UGCG* and *B3GALT4* was higher in Glutamatergic neuronal cultures and *ST8SIA1* and *ST8SIA5* higher in Peri.4U (Figure 2C). In order to confirm the expression of some of these genes and gangliosides, immunostainings were conducted on the 4 models after 14 days in culture.

**FIGURE 3:**
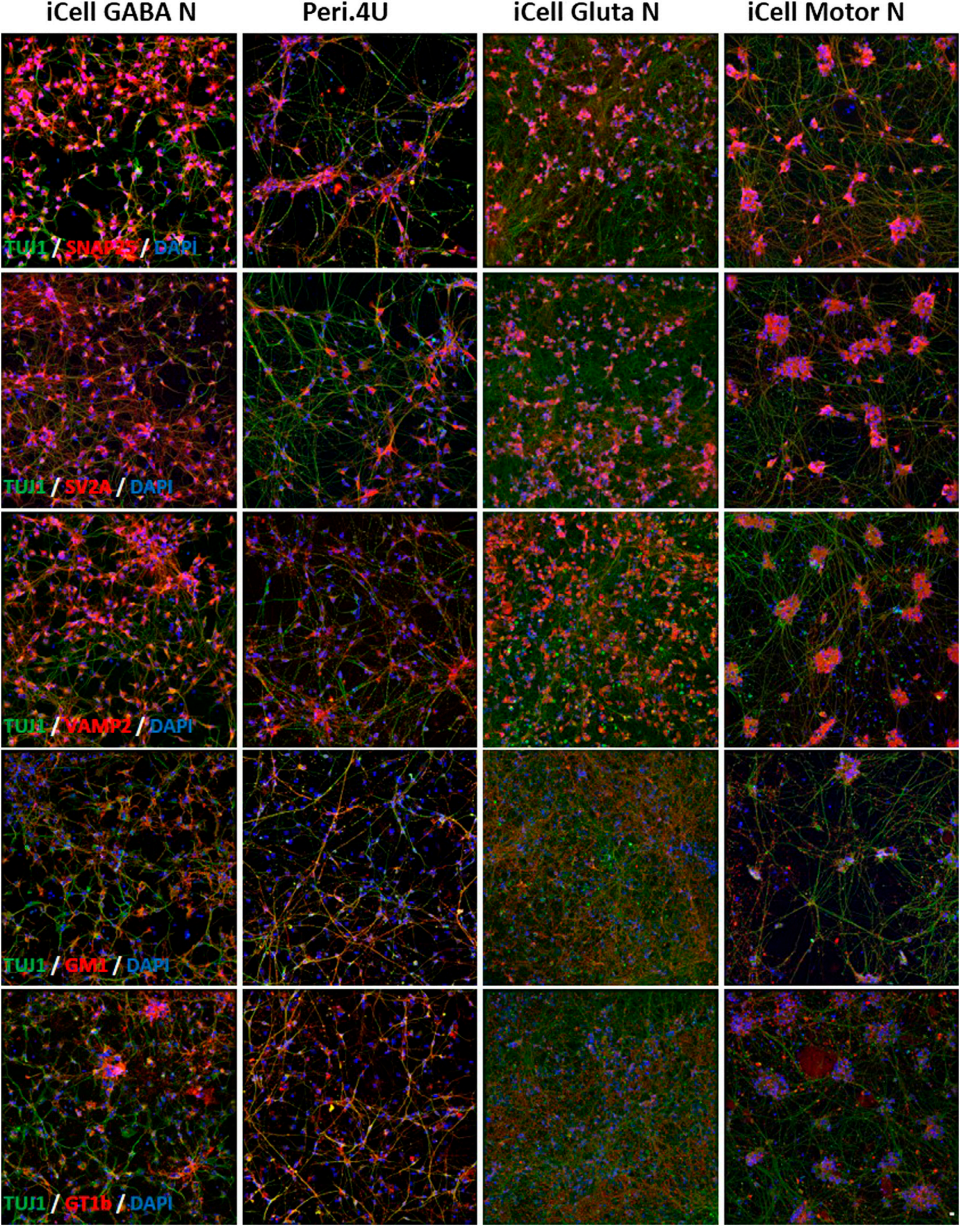
Immunolabelings of hiPSC-derived Neurons at day 15 after thawing: **(A–E)** TUJ1 (green) and **(A)** SNAP25 (red); **(B)** SV2A (red); **(C)** VAMP2 (red); **(D)** GM1 (red) and **(E)** GT1b (red). Nuclei are stained in blue (DAPI). Scale bar: 25 μm.

Staining with specific antibodies for *SNAP25*, *VAMP2* and *SV2A* confirmed the high expression of these proteins on the 4 hiPSC-derived neuronal cultures (Figure 3). The expression of *GT1b* and *GM1*, 2 major gangliosides was confirmed in all 4 models (Figure 3).

All these data indicate that the four models express molecules involved in the different steps of BoNT intoxication process. Very interestingly, the expression level of the genes coding for BoNT receptors, SNAREs, TXR or even for enzymes involved in gangliosides biosynthesis are not the same amongst the four neuronal models tested and this could be translated in different sensitivity to toxins.

### Sensitivity of Different hiPSC-Derived Models to BoNT/A and BoNT/E

In order to determine whether the differential expression of genes encoding key proteins for BoNT activity might affect the sensitivity of the different hiPSC-derived neuronal models to BoNTs, the four neuronal cultures were exposed for 24 h to serial dilutions of recombinant BoNT/A and BoNT/E at 14 days after thawing. Western blot analyses of cell lysates were performed to measure the percentage of cleaved SNAP25.

**FIGURE 4:**
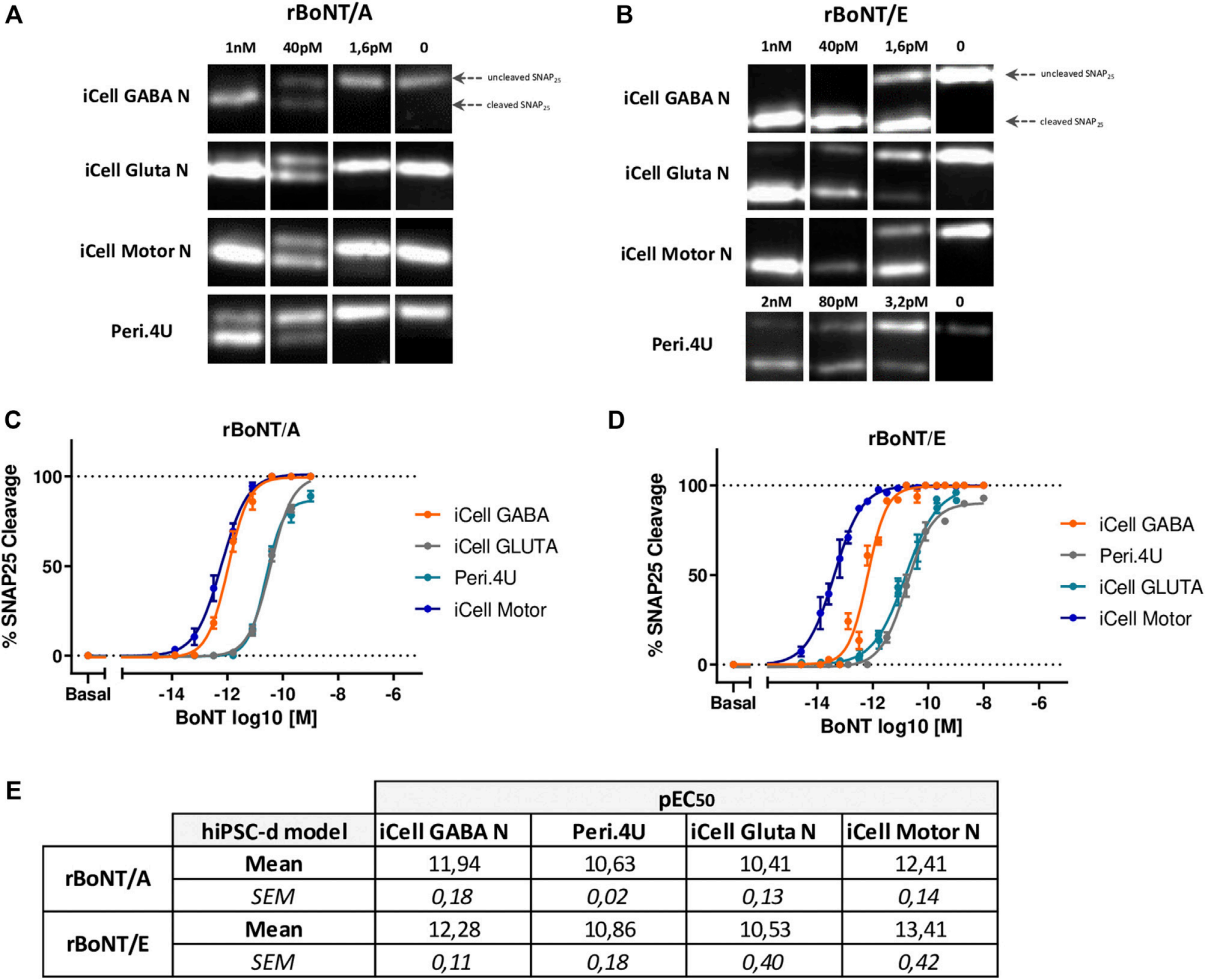
Sensitivity of the 4 models to rBoNT/A and rBoNT/E. **(A,B)**: Western Blots showing the cleavage of SNAP25 in the 4 different cellular models after treatment of BoNT/A **(A)** or BoNT/E **(B)** at different doses. **(C,D)** Dose response curves of SNAP25 cleavage for BoNT/A **(C)** and BoNT/E **(D)**. **(E)** Table of the potencies (pEC^50^) for BoNT/A and BoNT/E in each model; *n* = 3 for all models.

After 24 h of exposure to different doses of rBoNT/A and rBoNT/E, measurement of SNAP25 cleavage by Western Blot (Figures 4A,B) indicated a dose-response effect of both toxins in all 4 models (Figures 4C,D). Differences in pEC_50_ and maximum cleavage were identified between the models and toxins (Figures 4C–E). For rBoNT/A and rBoNT/E, iCell Motor Neurons were the most sensitive model with EC_50_ of 0.39 pM (10^−12.41^) and 0.04 pM (10^−13.41^) respectively. iCell GABA neurons represented the second most sensitive model with EC_50_ of 1.14 pM for rBoNT/A and 0.53 pM for rBoNT/E. Both Peri.4U and iCell Glutamatergic Neurons were less sensitive than the two other models. EC_50_ for Peri.4U are 23.71 pM for rBoNT/A and 13.70 pM for rBoNT/E. EC_50_ for Glutamatergic Neurons were 38.61 pM for rBoNT/A and 29.40 pM for rBoNT/E (Figure 4E).

These data indicate that Motor Neurons derived from hiPSC are one of the most sensitive models to rBoNT/A and the most sensitive to rBoNT/E highlighting the potential of this model for developing physiologically relevant models for determining the activity and characterizing the activity of BoNTs.

### Motor Neurons Exhibit Physiological Features and Functional Activity After Only 7 days of Maturation

In order to further analyze the gene profile of Motor Neurons derived from hiPSC, we performed a RNASeq unsupervized analysis of Motor Neurons maturated during 1, 14 and 28 days. In order to compare the different gene profiles, RNA from human spinal cord has been included in the analysis.

**FIGURE 5:**
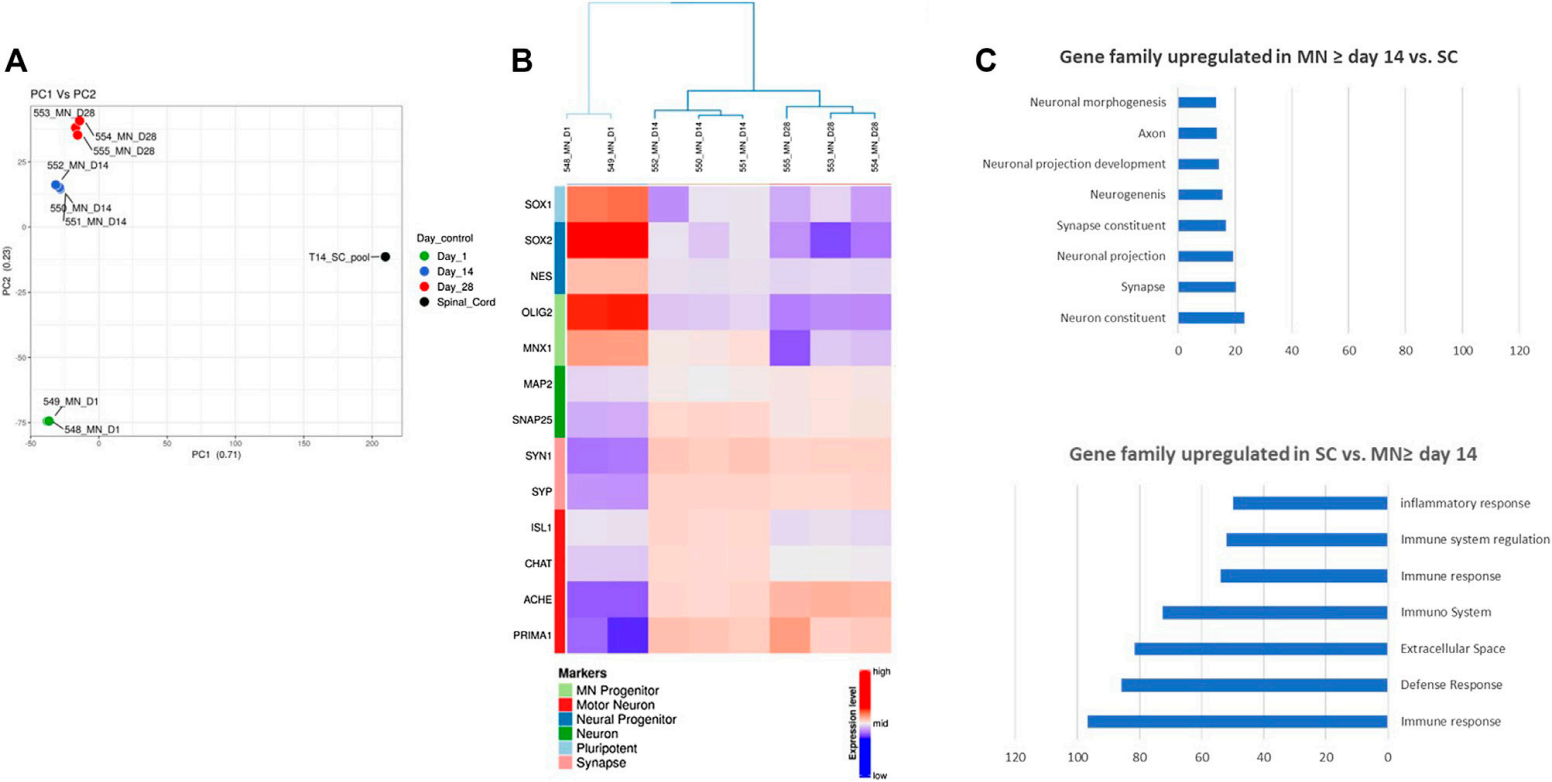
RNASeq analysis of hiPSC-derived Motor Neurons. **(A)** Principal Component Analysis showing distinct signatures at different time points during maturation of hiPSC-derived Motor neurons and Spinal cord RNA sample. **(B)** Heat map illustrating the time-course of Motor Neurons progenitors and mature Motor Neurons expression **(C)** Enrichment analysis showing classes of genes that are over-represented in Motor Neurons after 2 weeks of maturation compared to a human tissue sample (Spinal Cord) and classes of genes that are over-represented over-represented in human spinal cord compared to Motor Neurons after 2 weeks of maturation. Expressions are in ‐logp value. Only the top gene sets of the Gene Ontology collection are represented.

The Principal Component analysis (PCA) of Motor Neurons and Spinal Cord showed that hiPSC-derived Motor Neurons exhibited similar profiles within a same maturation stage and that profiles specific to each stage are different. The analysis indicated also that Motor Neurons, whatever the maturation stage, exhibited a distinct signature compared to Spinal Cord (Figure 5A). Furthermore, the profile of Motor Neurons at day 28 was closer to that of the profile of Motor Neurons maturated for 14 days than the profile after 1 day maturation, suggesting that the 2 first weeks in culture had a higher impact on the degree of maturation of hiPSC-derived Motor Neurons than weeks three and four (Figure 5A).

To further investigate this, we performed a hierarchical clustering and consensus clustering analysis. On the first maturation day, Motor Neurons expressed genes of pluripotency (*SOX1*), neural progenitors (*NES*, *SOX2*) and Motor Neurons progenitors (*OLIG2*, *MNX1*) whereas Motor Neurons at day 14 or day 28 of maturation expressed mostly neuronal markers (*MAP2*, *SNAP25*), markers of synaptic connection (SYN and SYP) and motor neuron markers (*ISL1*, *CHAT*, *ACHE*, and *PRIMA1*) (Figure 5B). Interestingly the levels of expression for *ISL1* and *CHAT* were higher after 14 days maturation than 28 days.

A differential expression analysis and a pathway enrichment analysis between hiPSC-derived Motor Neurons maturated more than 14 days and Spinal cord was performed. These analyses showed that Motor Neurons transcriptome was enriched with genes involved in neuronal development, neuronal function, synaptic maturation and function whereas Spinal Cord transcriptome was enriched with genes mainly involved in tissue physiology such as genes coding for proteins of the extracellular matrix or the inflammatory and immune response (Figure 5C).

Altogether, these data confirm that hiPSC-derived Motor Neurons express markers of authentic and functional Motor Neurons after 14 days of maturation.

**FIGURE 6:**
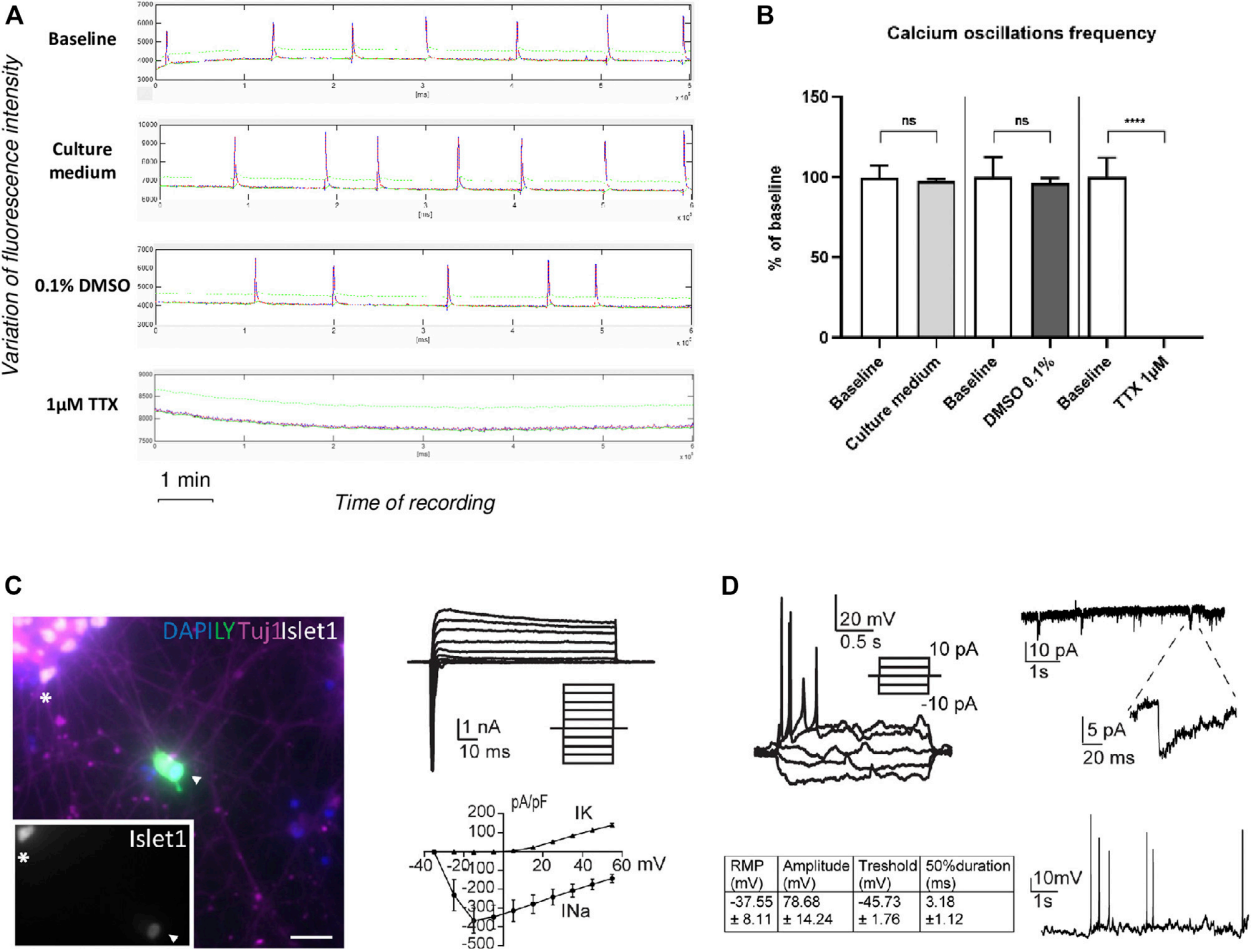
Functional characterization of Motor neurons. **(A)** Representatives traces of Ca2+ oscillations obtained by whole-well recording followed by FDSS. Waveformanalysis of Cal520 dye loaded 28 days old-iCell MN cultures. Cells were stained with Cal520 the day of the recording. Ca2+ oscillations were recorded before (i.e. baseline condition) and after addition of culture medium, 0.1% DMSO or 1 μM TTX. **(B)** Ca2+ oscillations frequency of iCell Motor Neurons after addition of culture medium, 0.1% DMSO or 1 μM TTX, compared to baseline condition without treatment. Data are represented as mean ± SEM. ANOVA with Sidak correction (****p < 0.0001, n.s. not significant). **(C)** Left: Patch-clamped iPSC-derived MN (arrowhead) filled with lucifer yellow (LY) and identified after recording by labelling for Islet1 (inset) and Tuj1. Scale bar 20 μm. Right: Voltage-clamp whole-cell current-voltage relationship. Top: representative trace and corresponding voltage steps; bottom: current densities plotted against voltage pulses. **(D)** Left, Top: Current-clamp whole-cell current-voltage relationship with current pulses applied. Bottom: table summarizing action potentials properties and resting membrane potential (RMP). Right: Synaptic inputs and zoom in on one event. Bottom: spontaneous firing of action potentials.

In order to assess if human Motor Neurons acquired functional properties, we performed both Ca^2+^ imaging using a Spinning Disk system and Ca^2+^ oscillations recording using a high content plate reader on Motor Neurons cultures. After 28 days in culture, Motor Neurons exhibited synchronized spontaneous Ca^2+^ oscillations that were recorded both using the Spinning Disk (Supplementary Figure S1) and whole-well Cal520 calcium imaging (Figure 6A) in basal condition. The addition of culture medium or DMSO 0,1% did not alter these oscillations (Figure 6A and Supplementary Figure S1). Conversely, addition of 1 µM of Tetrodotoxin (TTX), a strong Na^+^ channel blocker, completely abolished both Ca^2+^ signal (Supplementary Figure S1) and oscillation (Figures 6A,B).

These data demonstrate that Motor Neurons acquire functional properties in culture and are able to form connections.

To confirm the functional signature of Motor Neurons, we performed whole-cell recordings on Motor Neurons maturated after 14 days. Motor Neurons were identified by their dye-filled morphology and post-recordings immunocytochemistry (Figure 6C, Left, Islet1 and TUJ1 positive cell). Whole-cell voltage clamp recordings revealed the presence of voltage-activated sodium and potassium channels (Figure 6C right).

On day 14, 100% of the Motor Neurons (Islet1 and TUJ1 positive cells) fired action potentials in response to a 50 pA current injection (*n* = 5), with the threshold, shape and magnitude being typical for mature neurons (Figure 6D, Left). As previously described (Devlin et al., 2015), three types of Motor Neurons can be distinguished based on their response to current injections: single, adaptive and repetitive. Here, we mainly identified adaptive and repetitive Motor Neurons (Figure 6D and Supplementary Figure S2A). Moreover, all Motor Neurons received synaptic inputs and showed spontaneous action potentials, indicating a functional network (Figure 6D, Right).

Altogether our data demonstrate that after only 14 days in culture, Motor Neurons display electrophysiological properties of mature neurons and typical characteristics of functional Motor Neurons, such as receiving post-synaptic inputs and firing of action potentials.

### When Cultivated With Human Myotubes, Motor Neurons Form Functional NMJ Sensitive to BoNT

To evaluate the potential of hiPSC-derived Motor Neurons for generating a human *in vitro* model of the neuromuscular junction, we tested whether Motor Neurons could innervate human myotubes. To this aim human immortalized skeletal myoblasts were thawed and seeded into 96-well plates and grown in skeletal muscle growth medium for one day, and in differentiation medium to induce myoblast fusion and myotube formation.

**FIGURE 7:**
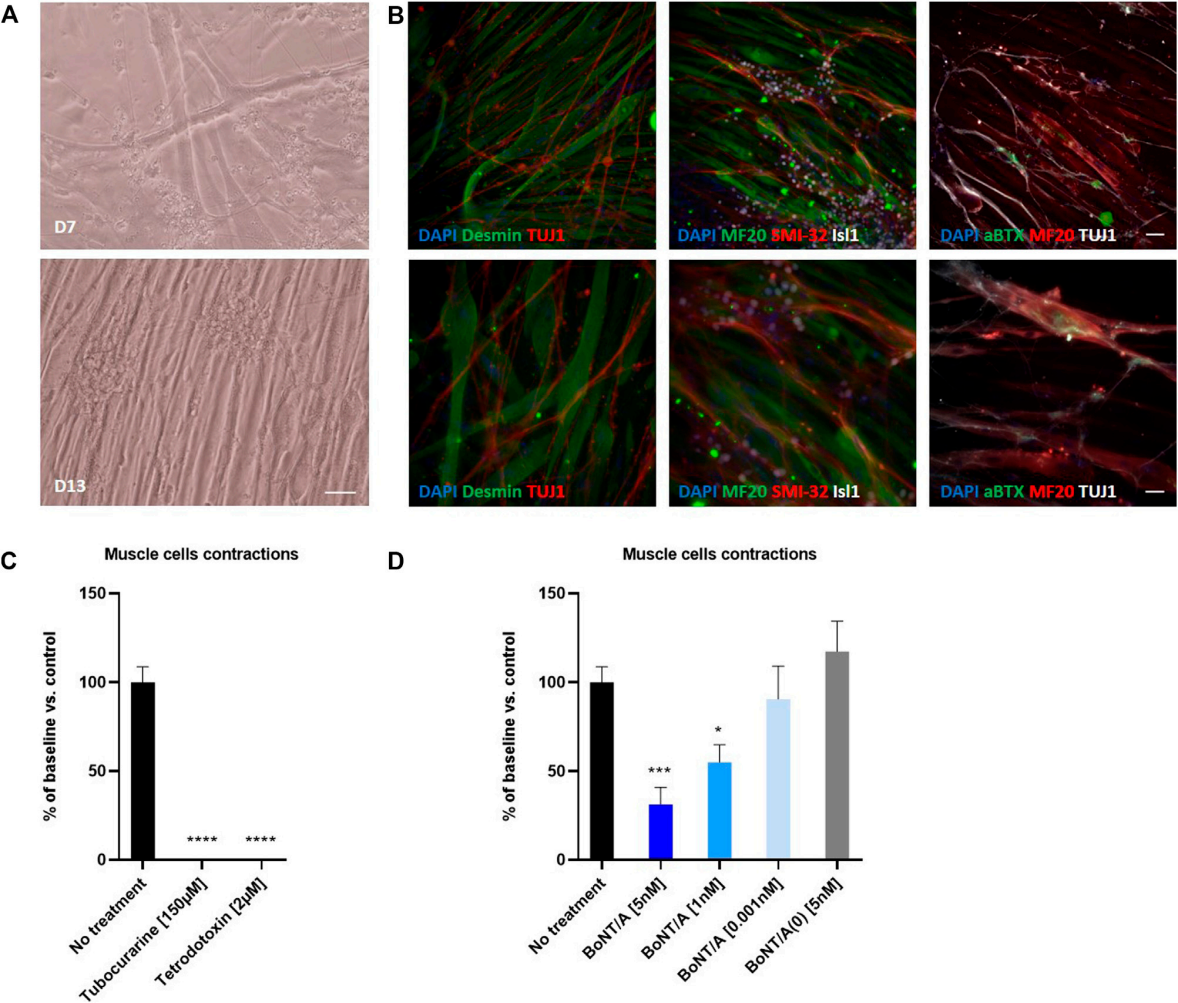
Coculture of human myotubes and human motor neurons. **(A)** Phase images of co-cultures maintained in P96 during 7 or 13 days. Scale bar: 25 µm. **(B)** Immunocytochemical characterization of NMJ formation. Muscular fibers are identified with Myosin/MF20 or Desmin, Motor Neurons neurites are identified with TUJ1 or SMI-32 and MN nuclei are identified with Islet 1 and Acetylcholine receptor with alpha-bungarotoxin. Nuclei are represented in blue (DAPI). Top: 20X images; scale bar: 50 µm. Bottom: 40X images; scale bar: 25 µm. **(C)** Effect of TTX and Tubocurarine on muscle cells contractions after 6 h of exposure. **(D)** Dose-response effects of BoNT/A on contraction of myotubes after 6 h of exposure. *p < 0.05; **p > 0.01; ***p < 0.001; ****p < 0.0001.

To generate the coculture system, Motor Neurons were thawed and seeded directly on the muscle fiber cultures after 1 day of maturation. After 15 days of coculture, large myotube fibers expressing desmin and a dense TUJ1 or SMI32 + neuronal network were observed suggesting that both cell types, neurons and myotubes, continued to mature within the coculture (Figures 7A,Bfig7). Immunostainings conducted at day 15 of maturation confirmed that viable Motor Neurons expressing islet1 and mature myotubes expressing Myosin can be identified in the coculture system (Figure 7B). In addition, clusters of αBTX-positive acetylcholine receptors were identified at the site of TUJ1-positive Motor Neurons suggesting the formation of NMJ between human Motor Neurons and human Myotubes (Figure 7B). In parallel, control immunostainings were performed on myotubes alone after 15 days of culture, confirming the specificity of stainings with the absence of neuronal markers expression (Supplementary Figure S3).

To evaluate whether or not the coculture system affected the electrophysiological properties of Motor Neurons, we performed a patch clamp analysis of Motor Neurons cultivated during 14 days in presence of myotubes. Similar to Motor Neurons recorded in monocultures, patched Motor Neurons in coculture fell into two categories when current pulses were applied: they adapted and only produced one or a few action potentials or they generated repetitive action potentials throughout the pulse (Supplementary Figure S2B,C). They also displayed the same functional characteristics such as receiving synaptic inputs and firing spontaneous action potentials (Supplementary Figure S2D).

We then evaluated the functionality of this neuromuscular coculture system by monitoring myotubes contractions using video-microscopy (Supplementary Figure S4). Myotube contractions were identified in 100% of wells where myotubes and motoneurons were co-cultivated. The number of myotube contractions per minute averaged 4.2 ± 0.35 (*n* = 9). Conversely, no contraction was identified in wells where myotubes were cultivated in absence of Motor Neurons (Supplementary Figure S4). Treatment of the co-cultures with 2 µM of TTX, a neuronal gated sodium channel inhibitor or 150 µM of tubocurarine, a muscular nicotinic acetylcholine receptor antagonist, abolished myotube contraction demonstrating the functionality of the system (Figure 7C and Supplementary Figure S4).

Finally, we assessed whether BoNT was able to inhibit myotube contractions in this system. To this end, cocultures of myotubes and Motor Neurons were exposed to different doses of BoNT/A and non-active BoNT/A (BoNT/A(0)) for 6 and 24 h. After 6 and 24 h of exposure, BoNT/A concentration-dependently reduced the number of myotube contractions. Thus, compared to control, the contractions were significantly reduced after 6 h to 31.33 ± 9.67 % (*n* = 9) and 55 ± 9.92 % (*n* = 9) for 5 and 1 nM of BoNT/A, respectively, whereas 0,001 nM BoNT/A was without significant effect (90.56 ± 18.57 % (*n* = 9) (Figure 7D). This inhibitory effect was even stronger after 24 h of exposure, because 5 nM of BoNT/A completely inhibited the contraction, and 1nM significantly reduced contractions to 17 ± 11.3 % (*n* = 9) of control, with 0.001 nM BoNT/A still being without significant effect (78.33 ± 28.04 % (*n* = 9) (Supplementary Figure S4E). Addition of 5 nM BoNT/A(0) did not modify the number of contractions (Figure 7D).

Altogether our data demonstrate that hiPSC-derived Motor Neurons when cultured in presence of human myotubes induce myotubes contractions and are sensitive to BoNTs.

## Discussion

The objective of this study was to investigate the potential of hiPSC-derived neuronal models to generate a robust and physiologically relevant *in vitro* platform for testing, characterizing and comparing different BoNT serotypes. These hiPSC-derived models include GABAergic neurons, which were the first hiPSC-derived model used for BoNT testing and remain the most commonly used; Motor neurons, which are the most physiologically relevant model for BoNT testing, and two additional models Peripheral neurons and Glutamatergic neurons that might represent BoNT targets for various indications of peripheral and central nervous system disorders.

In order to assess the potential of these hiPSC-derived models for BoNT testing, we first characterized in detail the four models analyzing their phenotypic identity at different time points of culture, up to four weeks after thawing, and analyzing the expressions of genes and proteins that are involved in BoNT mechanism of intoxication. Both time course gene expression analysis and immunostainings performed on all four models showed they express neuronal markers, synaptic markers and markers specific for their respective identity as early as day 7 after thawing. The analysis also showed that these models express all proteins involved in BoNT intoxication process demonstrating the potential of all four models for BoNT testing. In addition, the time course analysis revealed that the expression for all genes tested are already well established at day 7 after thawing suggesting that these four models can be used for BoNT testing as soon as 1 week after thawing. These results complete and enrich the data obtained by Pellett et al. that reported the expression of a panel of key genes and at one single time point (Pellett et al., 2019).

Also, in order to conduct an exhaustive study of the expression of SNAREs in models derived from hiPSC, we measured the expression of other SNAREs than those conventionally considered in the field of BoNTs; such as *SNAP23*, *VAMP5*, *VAMP7*, *VAMP8* and *YKT6*. First of all, we found that Total Brain (TB) expressed a high level of *VAMP1* and *VAMP2*; a moderate level of *SNAP23*, *VAMP3*, *VAMP4*, *YKT6* and *VAMP7* and a low level of *VAMP5* and *VAMP8* compared to *SNAP25* (Supplementary Figure S5). Not surprisingly, we found that the 4 models studied expressed a low level compared to Total Brain (TB) of *VAMP5* and *VAMP8*, SNAREs mainly associated with the plasma membrane and intracellular vesicles in muscle cells and required in regulated exocytosis in pancreatic acinar cells respectively (Zeng et al., 1998; Wang et al., 2004). More interestingly, we found that the four models expressed, a high level of *SNAP23*, *VAMP7* and *YKT6* when compared to TB.

SNAP23 is structurally and functionally similar to SNAP25. While SNAP25 expression is restricted to neurons, SNAP23 is expressed ubiquitously (Ravichandran et al., 1996; Wang et al., 1997). Several studies reported SNAP23 expression in different brain regions such as cortex, hippocampus, cerebellum and thalamus (Prescott and Chamberlain, 2011) as well as in neuronal cultures (Suh et al., 2010; Grassi et al., 2015). Subcellular expression of SNAP23 in dendritic spines, GABA and glutamatergic synapses and colocalization of SNAP23 with constituents of the postsynaptic density was also reported (Mandolesi et al., 2009; Bragina et al., 2010; Suh et al., 2010). SNAP23 functions in neurons include actions for initial axonal elongation and establishment of neuronal polarity (Grassi et al., 2015) as well as functional regulation of postsynaptic glutamate receptors (Suh et al., 2010).

Expression of *SNAP23* in our cultures might indicate presence of a rich network of neurites or elongation of axons and/or presence of glutamatergic and GABA synapses which correlates well with our observations and characterization that was performed.

VAMP7, also known as Tetanus neurotoxin insensitive - VAMP (TI-VAMP), is expressed in various mammalian tissues including brain and its expression can be detected in neuronal cultures (Rossi et al., 2004; Wang and Tang, 2006; Chaineau et al., 2009). In developing cerebral neurons, expression of VAMP7 was localized to axons and dendrites (Rossi et al., 2004). VAMP7 is involved in various important cellular functions including phagocytosis, mitosis, cell migration, membrane repair and growth as well as autophagosome biosynthesis (Rossi et al., 2004; Chaineau et al., 2009; Luzio et al., 2009; Moreau et al., 2011; Hesketh et al., 2014). In neurons, VAMP7 plays a key role in neuronal morphogenesis, neurite outgrowth and synaptic transmission (Coco et al., 1999; Alberts and Galli, 2003; Wang and Tang, 2006; Chaineau et al., 2009; Daste et al., 2015). A high level of *VAMP7* expression in hiPSC-derived neuronal cultures compared to TB could indicate a high level of synaptic connection or a higher level of neurite outgrowth than in TB where neuritogenesis is complete.

YKT6 or synaptobrevin analog YKT6 is highly enriched in brain especially cerebral cortex and hippocampus with very low level being detected in other tissues (Hasegawa et al., 2003; Rossi et al., 2004; Wang and Tang, 2006). YKT6 is involved in endoplasmic reticulum (ER) to Golgi transport, intra-Golgi, endosome–Golgi and vacuolar transport steps as well as in membrane fusion reactions that result in expansion and closure of the autophagosome membrane (McNew et al., 1997; Kweon et al., 2003; Fukasawa et al., 2004; Nair et al., 2011). The expression of *YKT6* in hiPSC-derived neuronal cultures suggests the presence of intracellular vesicle recycling and highlights the potential of these neuronal models to study the effect induced by the cleavage of YKT6 by BoNT/X (Zhang et al., 2017).

Treatment of these models with BoNT/A and BoNT/E indicated that all four models are sensitive to both BoNTs but exhibit different sensitivity. Interestingly, we found that Motor Neurons exhibit very high sensitivity to BoNTs demonstrating the high potential of this model for further assay development for testing and developing BoNTs.

RNAseq study conducted on Motor Neurons confirms that this model expresses authentic markers of Motor Neurons and share signature with adult cells from the spinal cord.

Furthermore, functionality of Motor Neurons has been characterized by electrophysiology and confirmed by Calcium mobilization assay. When co-cultivated with myotubes, Motor Neurons functionality is maintained, and Motor Neurons are able to innervate the myotubes, within a week, inducing contraction of myotubes. Addition of BoNT to the system induces dose-dependent reduction in the number of contractions.

So far, two studies have reported BoNT testing on monocultures of human iPSC-derived Motor Neurons (Pellett et al., 2019; Schenke et al., 2020). In both studies, the sensitivity of hiPSC-derived Motor neurons to BoNT/A or BoNT/E was high which is aligned with our findings. The study published by Johnson's lab evaluated BoNT/A EC_50_ in GABA Neurons at 0.2 U/well and in Motor Neurons at 0.006 U/well, which suggests that Motor Neurons would be 33-fold more sensitive to BoNT/A compared to GABA Neurons while in our study, the fold change in sensitivity between these two models was 2,9 in favor to Motor Neurons. In regard to BoNT/E, EC_50_ in GABA Neurons was 0.9U/well and Motor Neurons 0.02U/well in Johnson’s study which represent a difference of 45 fold of gain in potency for Motor Neurons while in our study the difference in sensitivity when comparing Motor Neurons and GABA was evaluated at 13.6 fold increase in Motor Neurons. Also, when comparing the sensitivity of Motor Neurons to BoNT/A and BoNT/E, Johnson’s study reported that Motor Neurons were more sensitive to BoNT/A compared to BoNT/E by a 3,3 factor. In our study, Motor Neurons sensitivity to BoNT/E was found to be 10 times higher than sensitivity to BoNT/A.

These discrepancies in the sensitivity comparisons reported by the two studies may be due to several reasons. First of all this could be linked to the different natures of BoNT used; Pellett and al. used BoNT produced by Clostridium while in our study recombinant BoNT produced by E.Coli was used. Also, in the Pellett’s study BoNT/E_3_ has been used whereas in our study it is BoNT/E_1-3,_ a variant of BoNT/E1 with 4 or 5 amino acid differences. And finally, durations of exposure to toxins practiced are not the same, 48 h in Pellett’s study and 24 h in ours.

Another study, published more recently, has also reported results of BoNT/A testing on hiPSC- derived Motor Neurons (Schenke et al., 2020). In that study, EC_50_ for BoNT/A was estimated at 0.046 pM while in our study, EC_50_ was estimated at 0.39 pM. This discrepancy could be explained by the differences in nature of BoNT, protocols for toxin treatment and in models used. In Schenke’s study, Motor Neurons were exposed to BoNT/A produced by Clostridium during 48 h while in our study cells were exposed to recombinant BoNT/A during 24 h. Also, Schenke et al. generated their own model of Motor Neurons from hiPSC following a published protocol (Du et al., 2015) while we used hiPSC-derived Motor Neurons commercially available. They estimated the % of islet1+ Motor Neurons to be around 50% while the purity of Motor Neurons in the model used for our study was estimated to be more than 80%. Using commercial Motor Neurons, we reported a maximum of SNAP25 cleavage of 100% whereas Schenke et al. reported a maximum of cleaved SNAP25 smaller.

Nevertheless, and despite the deviations reported, our study and these studies reported a sensitivity of Motor Neurons to toxin E either equal to or greater than toxin A. This result is different than the data previously reported by assays carried out using primary cultures of rat eSCN (Donald et al., 2018) where the sensitivity of toxin E was reported to be more than 30 times lower than toxin A. Very recently, assays conducted in healthy volunteers as part of an First-in-Man study of the toxin E reported an equivalent or even greater effect of toxin E compared to toxin A (Pons et al., 2019) showing that human iPSC-derived Motor Neurons would have predicted clinical effects of botulinum toxins better than the rat eSCN cell-based assay in this case. These data underline the importance and translational value of assays using hiPSC-Motor Neurons for the development of BoNTs.

Another key aspect of human iPSC-derived Motor Neurons is their functionality and ability to innervate myotubes allowing the development of more complex and physiologically relevant models for testing BoNTs such as the Neuro Muscular Junction.

So far, only one study has reported botulinum testing using an *in vitro* model of human NMJ (Santhanam et al., 2018). In that study, motor neurons and myoblasts were cultivated in two chambers of a device separated by micro-tunnels allowing axonal outgrowth to the muscle chamber. A Dose Response effect of Botox^®^ was reported on myotubes contraction induced after electrical stimulation of Motor Neurons which highlights the potential of NMJ system for BoNT testing. However, the system developed by Santhanam et al. requires the use of a specialised experimental setup, such as coculture chambers and an electrical stimulation device (Santhanam et al., 2018).

The system described in our study simply requires conventional cell culture plates and traditional observation devices.

Despite the differences in their implementation and use, the two systems described in Santhanam’s study and in this article demonstrate the potential of the NMJ *in vitro* human model for testing BoNTs. However, certain aspects of the two models remain problematic for more generalized and standardized use. First of all, recording myotube contractions, image processing and calculating the frequency of contractions are aspects that should be refined. One way to do this could be to use calcium imaging, either using calcium dyes or *in situ* expression of a calcium sensor, to make signal detection easier (Steinbeck et al., 2016; Sebille et al., 2017; Lin et al., 2019; Swartz et al., 2020). Another aspect which deserves to be optimized is the coupling between the exclusive stimulation of motor neurons and the recording of myotube contractions. This would allow to precisely control the stimulation of motor neurons, applying for example different profiles of stimulation and thus would allow to study the effect of BoNTs on the synapses activated with these different profiles specifically. To do this, one of the possibilities could be to implement motor neurons with an optogene that would be stimulated by light (Steinbeck et al., 2016; Lin et al., 2019; Swartz et al., 2020).

Despite many technological improvements in drug testing, there is today still a high percentage of drug attrition due to lack of efficacy (Meseguer-Ripolles et al., 2018). The lack of physiologically relevant and predictive cell-based assays is one of the major obstacles (Waring et al., 2015). HiPSCs hold tremendous promise for translational research in neurological diseases (Devine and Patani, 2017; Silva and Haggarty, 2019). Such neurological diseases include, for example, Parkinson’s disease for which new compounds hold a promise as future therapeutics (Brunetti et al., 2020). In this context hiPSC could provide a valuable testing system for direct and preconditional signal effects both in isolated cell types and as a complex system such as a model of the neuromuscular junction, with pre- and post-synaptic components. Furthermore, as shown here, hiPSC provide a valuable model where to test SNARE function and new compounds impairing the SNARE complex, for example polyphenols, could be benchmarked against BoNTs. In regard to the development of BoNT therapeutics, rodent embryonic Spinal Cord Neurons (eSCN) are a widely used *in vitro* assay. There are however drawbacks to using this approach such as the use of a substantial number of animals, their labour-intensive isolation, the rapid loss of cell phenotype, significant batch-to-batch variation and their potential lack of predictability due to their animal origin (Pellett, 2012).

In conclusion, our study described the potential of four hiPSC-derived models to characterize and test BoNTs. By demonstrating the potential of hiPSC-derived Motor Neurons to generate a model of NMJ sensitive to toxin, our study also provides the demonstration that hiPSC -derived Motor Neurons could represent a major asset in improving the translational value of preclinical data on BoNTs. In addition, these new hiPSC-based models could also help the development of other novel therapeutics in the area of movement disorders.

## Data Availability Statement

The datasets presented in this study can be found in online repositories. The names of the repository/repositories and accession number(s) can be found in the article/Supplementary Material. The data can be accessed at the ENA - PRJEB41360

## Author Contributions

CN conceived the study. JD, SR, ER, and CB performed the generation of hiPSC-derived neurons cultures, performed BoNT treatments, immunostainings and analysed the data. HG performed the electrophysiological recordings and analysed the data. JL, ER and CB generated all data on the human NMJ system. CN, ER, and JK wrote the manuscript.

## Conflict of Interest

The authors declare that the research was conducted in the absence of any commercial or financial relationships that could be construed as a potential conflict of interest. All authors are Ipsen employees.

## References

[B1] Alberts P. Galli T. (2003). The cell outgrowth secretory endosome (COSE): a specialized compartment involved in neuronal morphogenesis. Biol. Cell 95, 419–424. 10.1016/S0248-4900(03)00074-1 14597259

[B98] Beard M. (2014). “Translocation, entry into the cell,” in *Molecular aspects of Botulinum Neurotoxin*. Editor K. A. Foster (New York, NY: Springer New York), 151–170. 10.1007/978-1-4614-9454-6_7

[B2] Benoit R. M. Frey D. Hilbert M. Kevenaar J. T. Wieser M. M. Stirnimann C. U. (2014). Structural basis for recognition of synaptic vesicle protein 2C by botulinum neurotoxin A. Nature 505, 108–111. 10.1038/nature12732 24240280

[B3] Bianchi F. Malboubi M. Li Y. George J. H. Jerusalem A. Szele F. (2018). Rapid and efficient differentiation of functional motor neurons from human iPSC for neural injury modelling. Stem Cell Res. 32, 126–134. 10.1016/j.scr.2018.09.006 30278374

[B4] Blasi J. Chapman E. R. Yamasaki S. Binz T. Niemann H. Jahn R. (1993). Botulinum neurotoxin C1 blocks neurotransmitter release by means of cleaving HPC-1/syntaxis. EMBO J. 12, 4821–4828. 7901002 10.1002/j.1460-2075.1993.tb06171.x PMC413934

[B5] Bragina L. Giovedì S. Barbaresi P. Benfenati F. Conti F. (2010). Heterogeneity of glutaminergic and GABAergic release machinery in cerebral cortex: analysis of synaptogyrin, vesicle-associated membrane protein, and syntaxis. Neuroscience 165, 934–943. 10.1016/j.neuroscience.2009.11.009 19909789

[B6] Brunetti G. Di Rosa G. Scuto M. Leri M. Stefani M. Schmitz-Linneweber C. (2020). Healthspan maintenance and prevention of Parkinson’s-like phenotypes with hydroxytyrosol and oleuropein aglycone in *C. elegans* . Int. J. Mol. Sci. 21, 2588 10.3390/ijms21072588 PMC7178172 32276415

[B7] Chaddock J. (2012). “Transforming the domain structure of botulinum neurotoxins into novel therapeutics,” in botulinum neurotoxins. Editors Rummel A. Binz T. (Berlin, Heidelberg: Springer), 287–306. 10.1007/978-3-662-45790-0_13 23239358

[B8] Chaineau M. Danglot L. Galli T. (2009). Multiple roles of the vesicular-SNARE TI-VAMP in post-Golgi and endosomal trafficking. FEBS Lett. 583, 3817–3826. 10.1016/j.febslet.2009.10.026 19837067

[B9] Charbord J. Poydenot P. Bonnefond C. Feyeux M. Casagrande F. Brinon B. (2013). High throughput screening for inhibitors of REST in neural derivatives of human embryonic stem cells reveals a chemical compound that promotes expression of neuronal genes: HTS for REST Inhibitors in Human NSCs. Stem Cell. 31, 1816–1828. 10.1002/stem.1430 23712629

[B10] Coco S. Raposo G. Martinez S. Fontaine J.-J. Takamori S. Zahraoui A. (1999). Subcellular localization of tetanus neurotoxin-insensitive vesicle-associated membrane protein (VAMP)/VAMP7 in neuronal cells: evidence for a novel membrane compartment. J. Neurosci. 19, 9803–9812. 10.1523/JNEUROSCI.19-22-09803.1999 10559389 PMC6782963

[B11] Daste F. Galli T. Tareste D. (2015). Structure and function of longin SNAREs. J. Cell Sci. 128, 4263–4272. 10.1242/jcs.178574 26567219

[B12] Devine H. Patani R. (2017). The translational potential of human induced pluripotent stem cells for clinical neurology: the translational potential of hiPSCs in neurology. Cell Biol. Toxicol. 33, 129–144. 10.1007/s10565-016-9372-7 27915387 PMC5325844

[B99] Devlin A.-C. Burr K. Borooah S. Foster J. D. Cleary E. M. Geti I. (2015). Human iPSC-derived motoneurons harbouring TARDBP or C9ORF72 ALS mutations are dysfunctional despite maintaining viability. Nat. Commun. 6, 5999 10.1038/ncomms6999 25580746 PMC4338554

[B13] Donald S. Elliott M. Gray B. Hornby F. Lewandowska A. Marlin S. (2018). A comparison of biological activity of commercially available purified native botulinum neurotoxin serotypes A1 to F1 *in vitro*, *ex vivo*, and *in vivo* . Pharmacol. Res. Perspect. 6, e00446 10.1002/prp2.446 30519475 PMC6261930

[B14] Dong M. (2006). SV2 is the protein receptor for botulinum neurotoxin A. Science 312, 592–596. 10.1126/science.1123654 16543415

[B15] Dong M. Liu H. Tepp W. H. Johnson E. A. Janz R. Chapman E. R. (2008). Glycosylated SV2A and SV2B mediate the entry of botulinum neurotoxin E into neurons. Mol. Biol. Cell 19, 5226–5237. 10.1091/mbc.e08-07-0765 18815274 PMC2592654

[B16] Du Z.-W. Chen H. Liu H. Lu J. Qian K. Huang C.-L. (2015). Generation and expansion of highly pure motor neuron progenitors from human pluripotent stem cells. Nat. Commun. 6, 6626 10.1038/ncomms7626 25806427 PMC4375778

[B17] Egawa N. Kitaoka S. Tsukita K. Naitoh M. Takahashi K. Yamamoto T. (2012). Drug screening for ALS using patient-specific induced pluripotent stem cells. Sci. Transl. Med. 4, 145ra104 10.1126/scitranslmed.3004052 22855461

[B18] Elliott M. Favre-Guilmard C. Liu S. M. Maignel J. Masuyer G. Beard M. (2019). Engineered botulinum neurotoxin B with improved binding to human receptors has enhanced efficacy in preclinical models. Sci. Adv. 5, eaau7196 10.1126/sciadv.aau7196 30746458 PMC6357751

[B19] Fonfria E. (2018). “Botulinum neurotoxin: a multifunctional protein for the development of new therapeutics,” in neurotoxins. Editor McDuffie J. E., London: InTech 10.5772/intechopen.69433

[B20] Fonfria E. Elliott M. Beard M. Chaddock J. Krupp J. (2018a). Engineering botulinum toxins to improve and expand targeting and SNARE cleavage activity. Toxins 10, 278 10.3390/toxins10070278 PMC6071219 29973505

[B21] Fonfria E. Maignel J. Lezmi S. Martin V. Splevins A. Shubber S. (2018b). The expanding therapeutic utility of botulinum neurotoxins. Toxins 10, 208 10.3390/toxins10050208 PMC5983264 29783676

[B22] Foran P. Lawrence G. W. Shone C. C. Foster K. A. Dolly J. O. (1996). Botulinum neurotoxin C1 cleaves both syntaxis and SNAP-25 in intact and permeabilized chromaffin cells: correlation with its blockade of catecholamine release. Biochemistry 35, 2630–2636. 10.1021/bi9519009 8611567

[B23] Foster K. A. (2014a). Molecular aspects of botulinum neurotoxin. New York, NY: Springer 10.1007/978-1-4614-9454-6

[B24] Foster K. A. (2014b). “Overview and history of botulinum neurotoxin research,” in Molecular aspects of botulinum neurotoxin. Editor Foster K. A. (New York, NY: Springer), 1–7. 10.1007/978-1-4614-9454-6_1

[B25] Fukasawa M. Varlamov O. Eng W. S. Sollner T. H. Rothman J. E. (2004). Localization and activity of the SNARE Ykt6 determined by its regulatory domain and palmitoylation. Proc. Natl. Acad. Sci. U.S.A. 101, 4815–4820. 10.1073/pnas.0401183101 15044687 PMC387331

[B26] Gautier H. O. B. Evans K. A. Volbracht K. James R. Sitnikov S. Lundgaard I. (2015). Neuronal activity regulates remyelination via glutamate signalling to oligodendrocyte progenitors. Nat. Commun. 6, 8518 10.1038/ncomms9518 26439639 PMC4600759

[B27] Gordon J. Amini S. White M. K. (2013). “General overview of neuronal cell culture,” in neuronal cell culture. Editors Amini S. White M. K. (Totowa, NJ: Humana Press), 1–8. 10.1007/978-1-62703-640-5_1

[B28] Grassi D. Plonka F. B. Oksdath M. Guil A. N. Sosa L. J. Quiroga S. (2015). Selected SNARE proteins are essential for the polarized membrane insertion of igf-1 receptor and the regulation of initial axonal outgrowth in neurons. Cell Discov 1, 15023 10.1038/celldisc.2015.23 27462422 PMC4860833

[B29] Gribaudo S. Tixador P. Bousset L. Fenyi A. Lino P. Melki R. (2019). Propagation of α-synuclein strains within human reconstructed neuronal network. Stem Cell Rep 12, 230–244. 10.1016/j.stemcr.2018.12.007 PMC6372945 30639210

[B100] Hamark C. Berntsson R. P.-A. Masuyer G. Henriksson L. M. Gustafsson R. Stenmark P. (2017). Glycans confer specificity to the recognition of ganglioside receptors by botulinum neurotoxin A. J. Am. Chem. Soc. 139, 218–230. 10.1021/jacs.6b09534 27958736

[B30] Hasegawa H. Zinsser S. Rhee Y. Vik-Mo E. O. Davanger S. Hay J. C. (2003). Mammalian Ykt6 is a neuronal SNARE targeted to a specialized compartment by its profilin-like amino terminal domain. Mol. Biol. Cell 14, 698–720. 10.1091/mbc.e02-09-0556 12589064 PMC150002

[B31] Hesketh G. G. Pérez-Dorado I. Jackson L. P. Wartosch L. Schäfer I. B. Gray S. R. (2014). VARP is recruited on to endosomes by direct interaction with retromer, where together they function in export to the cell surface. Dev. Cell 29, 591–606. 10.1016/j.devcel.2014.04.010 24856514 PMC4059916

[B32] Hooker A. Palan S. Beard M. (2016). Recombinant botulinum neurotoxin serotype A1 (SXN102342): protein engineering and process development. Toxicon 123, S40 10.1016/j.toxicon.2016.11.113

[B33] Karalewitz A. P.-A. Fu Z. Baldwin M. R. Kim J.-J. P. Barbieri J. T. (2012). Botulinum neurotoxin serotype C associates with dual ganglioside receptors to facilitate cell entry. J. Biol. Chem. 287, 40806–40816. 10.1074/jbc.M112.404244 23027864 PMC3504792

[B34] Keller J. E. Cai F. Neale E. A. (2004). Uptake of botulinum neurotoxin into cultured neurons. Biochemistry 43, 526–532. 10.1021/bi0356698 14717608

[B35] Kirkeby A. Grealish S. Wolf D. A. Nelander J. Wood J. Lundblad M. (2012). Generation of regionally specified neural progenitors and functional neurons from human embryonic stem cells under defined conditions. Cell Rep. 1, 703–714. 10.1016/j.celrep.2012.04.009 22813745

[B36] Kriks S. Shim J.-W. Piao J. Ganat Y. M. Wakeman D. R. Xie Z. (2011). Dopamine neurons derived from human ES cells efficiently engraft in animal models of Parkinson’s disease. Nature 480, 547–551. 10.1038/nature10648 22056989 PMC3245796

[B37] Kweon Y. Rothe A. Conibear E. Stevens T. H. (2003). Ykt6p is a multifunctional yeast R-SNARE that is required for multiple membrane transport pathways to the vacuole. Mol. Biol. Cell 14, 1868–1881. 10.1091/mbc.e02-10-0687 12802061 PMC165083

[B38] Lin C.-Y. Yoshida M. Li L.-T. Ikenaka A. Oshima S. Nakagawa K. (2019). iPSC-derived functional human neuromuscular junctions model the pathophysiology of neuromuscular diseases. JCI Insight 4, e124299 10.1172/jci.insight.124299 PMC6795289 31534050

[B39] Little D. Ketteler R. Gissen P. Devine M. J. (2019). Using stem cell–derived neurons in drug screening for neurological diseases. Neurobiol. Aging 78, 130–141. 10.1016/j.neurobiolaging.2019.02.008 30925301

[B40] Liu S. Yin N. Faiola F. (2017). Prospects and Frontiers of stem cell toxicology. Stem Cell. Dev. 26, 1528–1539. 10.1089/scd.2017.0150 PMC5661862 28874109

[B41] Luzio J. P. Parkinson M. D. J. Gray S. R. Bright N. A. (2009). The delivery of endocytosed cargo to lysosomes. Biochem. Soc. Trans. 37, 1019–1021. 10.1042/BST0371019 19754443

[B42] Mandolesi G. Vanni V. Cesa R. Grasselli G. Puglisi F. Cesare P. (2009). Distribution of the SNAP25 and SNAP23 synaptosomal-associated protein isoforms in rat cerebellar cortex. Neuroscience 164, 1084–1096. 10.1016/j.neuroscience.2009.08.067 19735702

[B43] Marteyn A. Maury Y. Gauthier M. M. Lecuyer C. Vernet R. Denis J. A. (2011). Mutant human embryonic stem cells reveal neurite and synapse formation defects in type 1 myotonic dystrophy. Cell Stem Cell 8, 434–444. 10.1016/j.stem.2011.02.004 21458401

[B44] Maury Y. Côme J. Piskorowski R. A. Salah-Mohellibi N. Chevaleyre V. Peschanski M. (2015). Combinatorial analysis of developmental cues efficiently converts human pluripotent stem cells into multiple neuronal subtypes. Nat. Biotechnol. 33, 89–96. 10.1038/nbt.3049 25383599

[B45] McNew J. A. Søgaard M. Lampen N. M. Machida S. Ye R. R. Lacomis L. (1997). Ykt6p, a prenylated SNARE essential for endoplasmic reticulum-golgi transport. J. Biol. Chem. 272, 17776–17783. 10.1074/jbc.272.28.17776 9211930

[B46] Meseguer-Ripolles J. Khetani S. R. Blanco J. G. Iredale M. Hay D. C. (2018). Pluripotent stem cell-derived human tissue: platforms to evaluate drug metabolism and safety. AAPS J. 20, 20 10.1208/s12248-017-0171-8 PMC5804345 29270863

[B47] Montal M. (2010). Botulinum neurotoxin: a marvel of protein design. Annu. Rev. Biochem. 79, 591–617. 10.1146/annurev.biochem.051908.125345 20233039

[B48] Montecucco C. (1986). How do tetanus and botulinum toxins bind to neuronal membranes?. Trends Biochem. Sci. 11, 314–317. 10.1016/0968-0004(86)90282-3

[B49] Moreau K. Ravikumar B. Renna M. Puri C. Rubinsztein D. C. (2011). Autophagosome precursor maturation requires homotypic fusion. Cell 146, 303–317. 10.1016/j.cell.2011.06.023 21784250 PMC3171170

[B50] Nair U. Jotwani A. Geng J. Gammoh N. Richerson D. Yen W.-L. (2011). SNARE proteins are required for macroautophagy. Cell 146, 290–302. 10.1016/j.cell.2011.06.022 21784249 PMC3143362

[B51] Nicoleau C. De Lamotte J. D. Raban E. Boude E. Noirmain F. Krupp J. (2018a). Assessment of multiple hiPSC-derived models for botulinum neurotoxin testing. Toxicon 156, S85 10.1016/j.toxicon.2018.11.206

[B52] Nicoleau C. Donald S. Pons L. De Lamotte J. D. Raban E. Fonfria E. (2018b). Translational value of hiPSC-derived models for botulinum neurotoxin research. Toxicon 156, S84–S85. 10.1016/j.toxicon.2018.11.205

[B53] Nicoleau C. Varela C. Bonnefond C. Maury Y. Bugi A. Aubry L. (2013). Embryonic stem cells neural differentiation qualifies the role of Wnt/β-Catenin signals in human telencephalic specification and regionalization: human ESC telencephalic differentiation. Stem Cell. 31, 1763–1774. 10.1002/stem.1462 23818270

[B54] Peck M. Smith T. Anniballi F. Austin J. Bano L. Bradshaw M. (2017). Historical perspectives and guidelines for botulinum neurotoxin subtype nomenclature. Toxins 9, 38 10.3390/toxins9010038 PMC5308270 28106761

[B55] Pellett S. (2012). in “Progress in cell based assays for botulinum neurotoxin detection,” in botulinum neurotoxins. Editors Rummel A. Binz T. (Berlin, Heidelberg: Springer), 257–285. 10.1007/978-3-662-45790-0_12 PMC3644986 23239357

[B56] Pellett S. Schwartz M. P. Tepp W. H. Josephson R. Scherf J. M. Pier C. L. (2015a). Human induced pluripotent stem cell derived neuronal cells cultured on chemically-defined hydrogels for sensitive in vitro detection of botulinum neurotoxin. Sci. Rep. 5, 14566 10.1038/srep14566 26411797 PMC4585966

[B57] Pellett S. Tepp W. H. Scherf J. M. Pier C. L. Johnson E. A. (2015b). Activity of botulinum neurotoxin type D (strain 1873) in human neurons. Toxicon 101, 63–69. 10.1016/j.toxicon.2015.04.015 25937339 PMC4458207

[B58] Pellett S. Tepp W. H. Johnson E. A. (2019). Botulinum neurotoxins A, B, C, E, and F preferentially enter cultured human motor neurons compared to other cultured human neuronal populations. FEBS Lett. 593, 2675–2685. 10.1002/1873-3468.13508 31240706 PMC7751886

[B59] Peng L. Berntsson R. P.-A. Tepp W. H. Pitkin R. M. Johnson E. A. Stenmark P. (2012). Botulinum neurotoxin D-C uses synaptotagmin I and II as receptors, and human synaptotagmin II is not an effective receptor for type B, D-C and G toxins. J. Cell Sci. 125, 3233–3242. 10.1242/jcs.103564 22454523 PMC4067266

[B60] Peng L. Tepp W. H. Johnson E. A. Dong M. (2011). Botulinum neurotoxin D uses synaptic vesicle protein SV2 and gangliosides as receptors. PLoS Pathog. 7, e1002008 10.1371/journal.ppat.1002008 21483489 PMC3068998

[B61] Pons L. Vilain C. Volteau M. Picaut P. (2019). Safety and pharmacodynamics of a novel recombinant botulinum toxin E (rBoNT-E): results of a phase 1 study in healthy male subjects compared with abobotulinumtoxinA (Dysport®). J. Neurol. Sci. 407, 116516 10.1016/j.jns.2019.116516 31655410

[B62] Prescott G. R. Chamberlain L. H. (2011). Regional and developmental brain expression patterns of SNAP25 splice variants. BMC Neurosci. 12, 35 10.1186/1471-2202-12-35 21526988 PMC3104942

[B63] Purkiss J. R. Friis L. M. Doward S. Quinn C. P. (2001). Clostridium botulinum neurotoxins act with a wide range of potencies on SH-SY5Y human neuroblastoma cells. Neurotoxicology 22, 447–453. 10.1016/S0161-813X(01)00042-0 11577803

[B64] Puttonen K. A. Ruponen M. Naumenko N. Hovatta O. H. Tavi P. Koistinaho J. (2015). Generation of functional neuromuscular junctions from human pluripotent stem cell lines. Front. Cell. Neurosci. 9, 473 10.3389/fncel.2015.00473 26696831 PMC4672046

[B65] Ravichandran V. Chawla A. Roche P. A. (1996). Identification of a novel syntaxin- and synaptobrevin/VAMP-binding protein, SNAP-23, expressed in non-neuronal tissues. J. Biol. Chem. 271, 13300–13303. 10.1074/jbc.271.23.13300 8663154

[B66] Restani L. Giribaldi F. Manich M. Bercsenyi K. Menendez G. Rossetto O. (2012). Botulinum neurotoxins A and E undergo retrograde Axonal transport in primary motor neurons. PLoS Pathog. 8, e1003087 10.1371/journal.ppat.1003087 23300443 PMC3531519

[B67] Rossetto O. Pirazzini M. Montecucco C. (2014). Botulinum neurotoxins: genetic, structural and mechanistic insights. Nat. Rev. Microbiol. 12, 535–549. 10.1038/nrmicro3295 24975322

[B68] Rossi V. Banfield D. Vacca M. Dietrich L. Ungermann C. Desposito M. (2004). Longins and their longin domains: regulated SNAREs and multifunctional SNARE regulators. Trends Biochem. Sci. 29, 682–688. 10.1016/j.tibs.2004.10.002 15544955

[B69] Rowe R. G. Daley G. Q. (2019). Induced pluripotent stem cells in disease modelling and drug discovery. Nat. Rev. Genet. 20, 377–388. 10.1038/s41576-019-0100-z 30737492 PMC6584039

[B70] Rummel A. (2015). The long journey of botulinum neurotoxins into the synapse. Toxicon 107, 9–24. 10.1016/j.toxicon.2015.09.009 26363288

[B71] Rummel A. (2016). Two feet on the membrane: uptake of clostridial neurotoxins,” in Uptake and Trafficking of protein toxins. Editor Barth H. (Cham: Springer International Publishing), 1–37. 10.1007/82_2016_48

[B72] Rummel A. Eichner T. Weil T. Karnath T. Gutcaits A. Mahrhold S. (2007). Identification of the protein receptor binding site of botulinum neurotoxins B and G proves the double-receptor concept. Proc. Natl. Acad. Sci. U.S.A. 104, 359–364. 10.1073/pnas.0609713104 17185412 PMC1716154

[B73] Sala L. van Meer B. J. Tertoolen L. G. J. Bakkers J. Bellin M. Davis R. P. (2018). MUSCLEMOTION: a versatile open software tool to quantify cardiomyocyte and cardiac muscle contraction in vitro and in vivo. Circ. Res. 122, e5–e16. 10.1161/CIRCRESAHA.117.312067 29282212 PMC5805275

[B74] Salinas S. Schiavo G. Kremer E. J. (2010). A hitchhiker’s guide to the nervous system: the complex journey of viruses and toxins. Nat. Rev. Microbiol. 8, 645–655. 10.1038/nrmicro2395 20706281

[B75] Santhanam N. Kumanchik L. Guo X. Sommerhage F. Cai Y. Jackson M. (2018). Stem cell derived phenotypic human neuromuscular junction model for dose response evaluation of therapeutics. Biomaterials 166, 64–78. 10.1016/j.biomaterials.2018.02.047 29547745 PMC5866791

[B76] Schenke M. Schjeide B.-M. Püschel G. P. Seeger B. (2020). Analysis of motor neurons differentiated from human induced pluripotent stem cells for the use in cell-based botulinum neurotoxin activity assays. Toxins 12, 276 10.3390/toxins12050276 PMC7291138 32344847

[B77] Schiavo G. Shone C. C. Bennett M. K. Scheller R. H. Montecucco C. (1995). Botulinum neurotoxin type C cleaves a single lys-ala bond within the carboxyl-terminal region of syntaxins. J. Biol. Chem. 270, 10566–10570. 10.1074/jbc.270.18.10566 7737992

[B78] Sebille S. Ayad O. Chapotte-Baldacci C.-A. Cognard C. Bois P. Chatelier A. (2017). Optogenetic approach for targeted activation of global calcium transients in differentiated C2C12 myotubes. Sci. Rep. 7, 11108 10.1038/s41598-017-11551-z 28894267 PMC5593883

[B79] Shi Y. Inoue H. Wu J. C. Yamanaka S. (2017). Induced pluripotent stem cell technology: a decade of progress. Nat. Rev. Drug Discov. 16, 115–130. 10.1038/nrd.2016.245 27980341 PMC6416143

[B80] Silva M. C. Haggarty S. J. (2019). Human pluripotent stem cell–derived models and drug screening in CNS precision medicine. *Ann. N. Y. Acad. Sci.*, nyas. 14012, 18–56. 10.1111/nyas.14012 PMC8193821 30875083

[B81] Simpson L. L. (1980). Kinetic studies on the interaction between botulinum toxin type A and the cholinergic neuromuscular junction. J. Pharmacol. Exp. Therapeut. 212, 16–21. 6243359

[B82] Steinbeck J. A. Jaiswal M. K. Calder E. L. Kishinevsky S. Weishaupt A. Toyka K. V. (2016). Functional connectivity under optogenetic control allows modeling of human neuromuscular disease. Cell Stem Cell 18, 134–143. 10.1016/j.stem.2015.10.002 26549107 PMC4707991

[B83] Stenmark P. Dong M. Dupuy J. Chapman E. R. Stevens R. C. (2010). Crystal structure of the botulinum neurotoxin type G binding domain: insight into cell surface binding. J. Mol. Biol. 397, 1287–1297. 10.1016/j.jmb.2010.02.041 20219474 PMC2928138

[B84] Strotmeier J. Willjes G. Binz T. Rummel A. (2012). Human synaptotagmin-II is not a high affinity receptor for botulinum neurotoxin B and G: increased therapeutic dosage and immunogenicity. FEBS Lett. 586, 310–313. 10.1016/j.febslet.2011.12.037 22265973

[B85] Suh Y. H. Terashima A. Petralia R. S. Wenthold R. J. Isaac J. T. R. Roche K. W. (2010). A neuronal role for SNAP-23 in postsynaptic glutamate receptor trafficking. Nat. Neurosci. 13, 338–343. 10.1038/nn.2488 20118925 PMC2861127

[B86] Swartz E. W. Shintani G. Wan J. Maffei J. S. Wang S. H. Miller B. L. (2020). Establishment of a human induced pluripotent stem cell-derived neuromuscular Co-culture under optogenetic control. Neuroscience. 10.1101/2020.04.10.036400

[B87] Takahashi K. Tanabe K. Ohnuki M. Narita M. Ichisaka T. Tomoda K. (2007). Induction of pluripotent stem cells from adult human fibroblasts by defined factors. Cell 131, 861–872. 10.1016/j.cell.2007.11.019 18035408

[B88] Tegenge M. A. Böhnel H. Gessler F. Bicker G. (2012). Neurotransmitter vesicle release from human model neurons (NT2) is sensitive to botulinum toxin A. Cell. Mol. Neurobiol. 32, 1021–1029. 10.1007/s10571-012-9818-2 22373696 PMC11498402

[B89] Wang C.-C. Ng C. P. Lu L. Atlashkin V. Zhang W. Seet L.-F. (2004). A role of VAMP8/endobrevin in regulated exocytosis of pancreatic acinar cells. Dev. Cell 7, 359–371. 10.1016/j.devcel.2004.08.002 15363411

[B90] Wang G. Witkin J. W. Hao G. Bankaitis V. A. Scherer P. E. Baldini G. (1997). Syndet is a novel SNAP-25 related protein expressed in many tissues. J. Cell Sci. 110 (Pt 4), 505–513. 9067602 10.1242/jcs.110.4.505

[B91] Wang Y. Tang B. L. (2006). SNAREs in neurons – beyond synaptic vesicle exocytosis (Review). Mol. Membr. Biol. 23, 377–384. 10.1080/09687860600776734 17060155

[B92] Waring M. J. Arrowsmith J. Leach A. R. Leeson P. D. Mandrell S. Owen R. M. (2015). An analysis of the attrition of drug candidates from four major pharmaceutical companies. Nat. Rev. Drug Discov. 14, 475–486. 10.1038/nrd4609 26091267

[B93] Whitemarsh R. C. M. Strathman M. J. Chase L. G. Stankewicz C. Tepp W. H. Johnson E. A. (2012). Novel application of human neurons derived from induced pluripotent stem cells for highly sensitive botulinum neurotoxin detection. Toxicol. Sci. 126, 426–435. 10.1093/toxsci/kfr354 22223483 PMC3307606

[B94] Willjes G. Mahrhold S. Strotmeier J. Eichner T. Rummel A. Binz T. (2013). Botulinum neurotoxin G binds synaptotagmin-II in a mode similar to that of serotype B: tyrosine 1186 and lysine 1191 cause its lower affinity. Biochemistry 52, 3930–3938. 10.1021/bi4003502 23647335

[B95] Yu J. Vodyanik M. A. Smuga-Otto K. Antosiewicz-Bourget J. Frane J. L. Tian S. (2007). Induced pluripotent stem cell lines derived from human somatic cells. Science 318, 1917–1920. 10.1126/science.1151526 18029452

[B96] Zeng Q. Subramaniam V. N. Wong S. H. Tang B. L. Parton R. G. Rea S. (1998). A novel synaptobrevin/VAMP homologous protein (VAMP5) is increased during in vitro myogenesis and present in the plasma membrane. Mol. Biol. Cell 9, 2423–2437. 10.1091/mbc.9.9.2423 9725904 PMC25509

[B97] Zhang S. Masuyer G. Zhang J. Shen Y. Lundin D. Henriksson L. (2017). Identification and characterization of a novel botulinum neurotoxin. Nat. Commun. 8, 14130 10.1038/ncomms14130 28770820 PMC5543303

