# Supplementary material for: hiPSC-Derived Neurons Provide a Robust and Physiologically Relevant In Vitro Platform to Test Botulinum Neurotoxins
Source: Front Pharmacol. 2021 Jan 14;11:617867. doi: 10.3389/fphar.2020.617867 (PMC7840483; doi:10.3389/fphar.2020.617867)
Supplement: Supplementary file 7 [file datasheet1.pdf]

frontiers

Supplementary Material

1 Supplementary Figure 1

A See video 1.mp4
B See video 2.avi

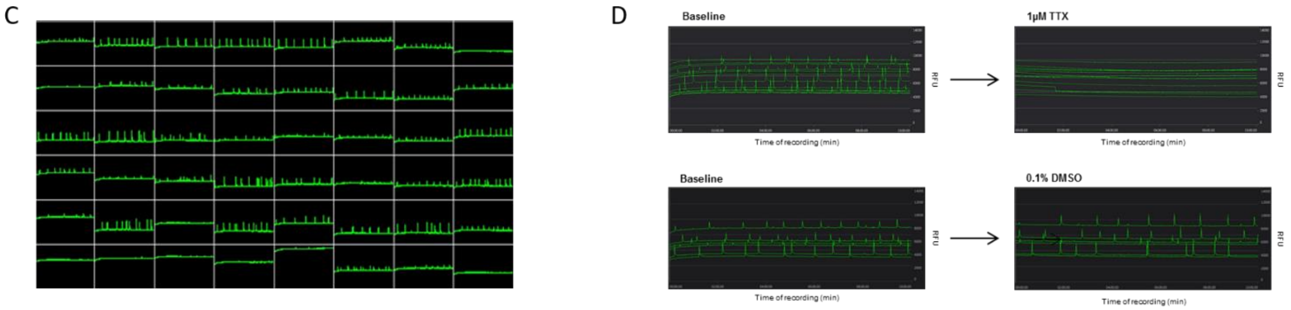

Supplementary Figure 1:
(A-B) Spontaneous and synchronous Ca2+ oscillations in iCell Motor neurons in basal condition and in response to TTX. (A) Representative timelapse microscope video of iCell Motor neurons after 28 days of maturation in response to 1µM TTX addition. Video illustrated the interruption of Ca2+ oscillations and the loss of fluorescence intensity from the cells following TTX addition. Spinning Disk system, scale bar 20X objective. (B) Mapping of the iCell Motor neurons 96-well plate being recorded at the FDSS 6000 in basal condition.
(C-D) Detection of fluorescent intensity changes in each well in response to recording buffer (basal condition). (C) Intracellular Ca2+ changes for 10 min in 96-wells plate (N=48 wells used for the assay) obtained by whole-well recording followed by FDSS 6000 of Cal520-AM dye loaded 28 days old-iCell Motor Neurons cultures, without manipulation of the media contained in each well (i.e. baseline). Ca2+ oscillations in each well occur at different rates and with a different timing. (D) Intracellular Ca2+ changes for 10 min in 96-wells plate (N=48 wells used for the assay) obtained by whole-well recording followed by FDSS 6000 of Cal520-AM dye loaded 28 days old-iCell Motor Neurons cultures, without manipulation of the media contained in each well (i.e. baseline), and after 1µM TTX or 0.1% DMSO addition. Ca2+ oscillations are inhibited after TTX treatment or unchanged after vehicle treatment.

2 Supplementary Figure 2

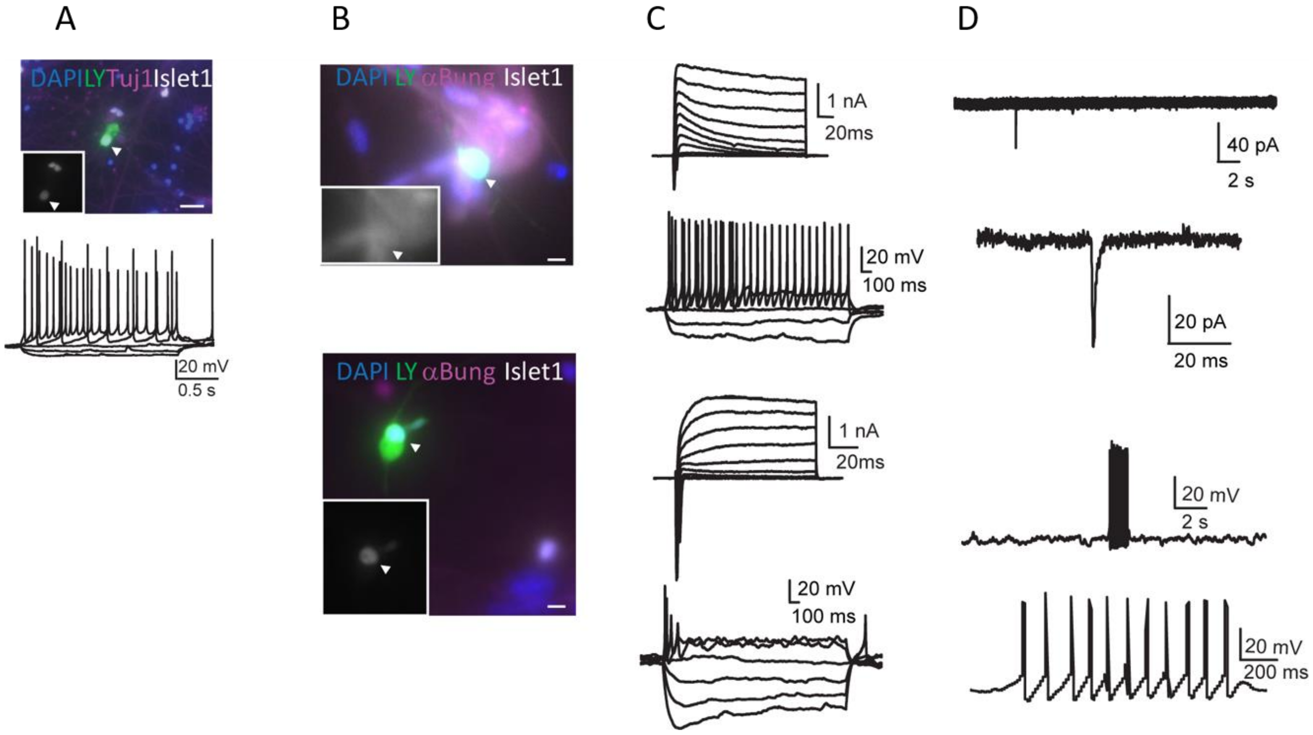

Supplementary Figure 2: Most common types of human Motor Neurons identified in mono and coculture are functional

(A) Patch-clamped iPSC-derived MN in monoculture filled with lucifer yellow (LY) and identified after recording by labelling for Islet1 (bottom, arrowhead) and Tuj1. Scale bar 50 µm.
Bottom: corresponding current-clamp current-voltage relationship from MN with adaptive firing (-20pA/+20pA)
(B) Patch-clamped iPSC-derived MN in co-culture filled with lucifer yellow (LY) and identified after recording by labelling for Islet1 (bottom, arrowhead) and α-bungarotoxin. Scale bar 10 µm.
(C) Corresponding current voltage relationship in Voltage-clamp (-35mV/+55mV) and current-clamp for 2 types of MNs: top continuous firing (-20pA/+20pA), bottom adaptive firing (-30pA/+20pA)
(D) Voltage-clamp recording of synaptic inputs, and zoom in. Bottom: Current clamp recording of spontaneous activity and zoom in.

3 Supplementary Figure 3

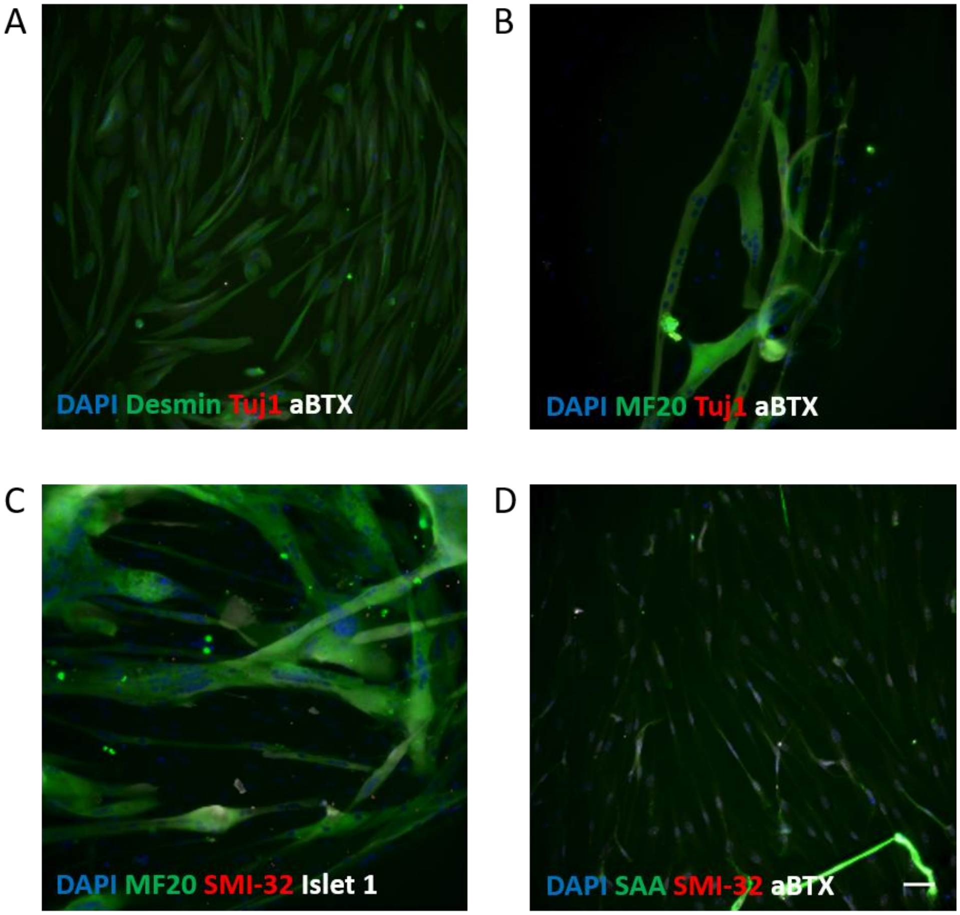

Supplementary Figure 3: Immunocytochemical images of myotubes alone after 14 days (20X, scale bar: 50µm) corresponding to negative controls. Nuclei are stained in blue, muscular fibers (Myosin/MF20, Desmin, SAA) in green, MN neurites (TUJ1, SMI-32) in red, alpha-bungarotoxin and MN nuclei (Islet 1) in white.
(A) DAPI / Desmin / TUJ1 / aBTX
(B) DAPI / MF20 / TUJ1 / aBTX
(C) DAPI / MF20 / SMI-32 / Islet 1
(D) DAPI / SAA / SMI-32 / aBTX

4 Supplementary Figure 4

A - See video 3.mp4
B - See video 4.mp4
C - See video 5.mp4
D - See video 6.mp4

E

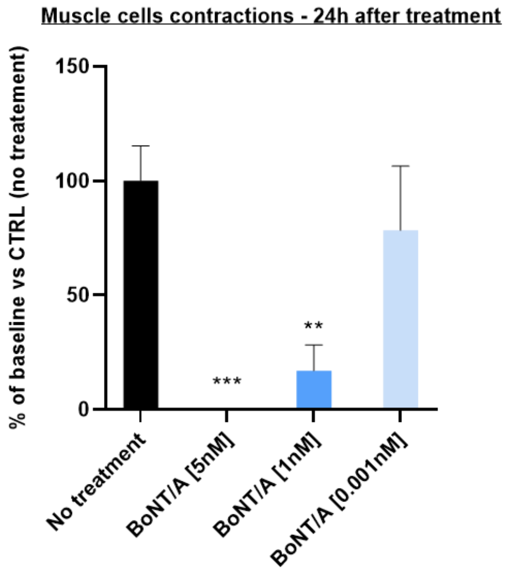

Supplementary Figure 4:
(A) video showing myotubes contraction in monoculture of myotubes in control condition
(B-D) video showing myotubes contraction in coculture of Motor Neurons and myotubes in control condition (B), after addition of 150µM of Tubocurarine (C) and after addition of 5nM BONT/A (D)
(E) Effect of BoNT/A on myotubes contraction frequency after 24 hours of treatment

5 Supplementary Figure 5

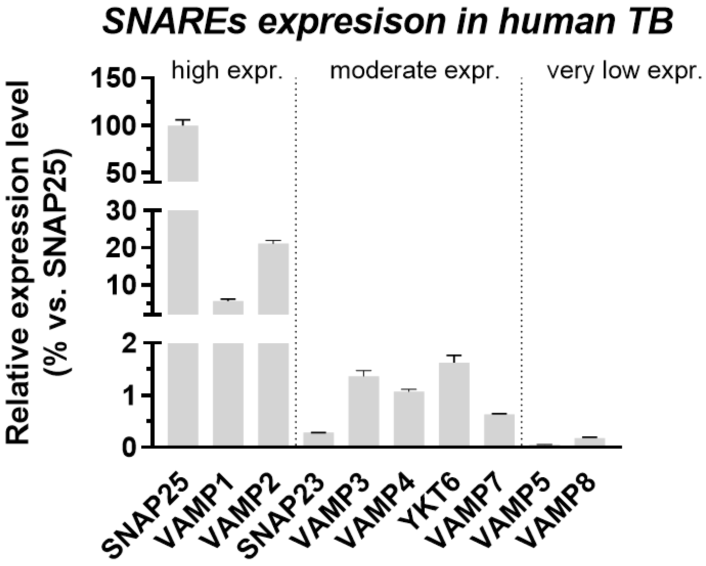

Supplementary Figure 5: Expression of the different BoNT SNARE substrates in human Total Brain.

Expression is normalized to GAPDH and to SNAP25 expression.
